# Allicin Bioavailability and Bioequivalence from Garlic Supplements and Garlic Foods

**DOI:** 10.3390/nu10070812

**Published:** 2018-06-24

**Authors:** Larry D. Lawson, Scott M. Hunsaker

**Affiliations:** Mérieux NutriSciences Corporate Office (Silliker, Inc.), 111 E. Wacker Dr. Ste. 2300, Chicago, IL 60601, USA; scott.huns@gmail.com

**Keywords:** allicin bioavailability, allicin metabolism, allyl methyl sulfide, alliin, *S*-allylcysteine, garlic supplements, cooked garlic, pickled garlic, black garlic, aged garlic extract

## Abstract

Allicin is considered responsible for most of the pharmacological activity of crushed raw garlic cloves. However, when garlic supplements and garlic foods are consumed, allicin bioavailability or bioequivalence (ABB) has been unknown and in question because allicin formation from alliin and garlic alliinase usually occurs after consumption, under enzyme-inhibiting gastrointestinal conditions. The ABB from 13 garlic supplements and 9 garlic foods was determined by bioassay for 13 subjects by comparing the area under the 32-h concentration curve of breath allyl methyl sulfide (AMS), the main breath metabolite of allicin, to the area found after consuming a control (100% ABB) of known allicin content: homogenized raw garlic. For enteric tablets, ABB varied from 36–104%, but it was reduced to 22–57% when consumed with a high-protein meal, due to slower gastric emptying. Independent of meal type, non-enteric tablets gave high ABB (80–111%), while garlic powder capsules gave 26–109%. Kwai garlic powder tablets, which have been used in a large number of clinical trials, gave 80% ABB, validating it as representing raw garlic in those trials. ABB did not vary with alliinase activity, indicating that only a minimum level of activity is required. Enteric tablets (high-protein meal) disintegrated slower in women than men. The ABB of supplements was compared to that predicted in vitro by the dissolution test in the United States Pharmacopeia (USP); only partial agreement was found. Cooked or acidified garlic foods, which have no alliinase activity, gave higher ABB than expected: boiled (16%), roasted (30%), pickled (19%), and acid-minced (66%). Black garlic gave 5%. The mechanism for the higher than expected ABB for alliinase-inhibited garlic was explored; the results for an alliin-free/allicin-free extract indicate a partial role for the enhanced metabolism of γ-glutamyl *S*-allylcysteine and *S*-allylcysteine to AMS. In conclusion, these largely unexpected results (lower ABB for enteric tablets and higher ABB for all other products) provide guidelines for the qualities of garlic products to be used in future clinical trials and new standards for manufacturers of garlic powder supplements. They also give the consumer an awareness of how garlic foods might compare to the garlic powder supplements used to establish any allicin-related health benefit of garlic.

## 1. Introduction

Garlic supplements, mainly dried and pulverized whole clove supplements, have been used in a large number of controlled clinical trials since the mid-1980s, focusing primarily on serum cholesterol and blood pressure [1,2,3]. The effects on blood pressure have been moderately consistent for hypertensive subjects, while the effects on serum lipids have been inconsistent, even for persons with high baseline cholesterol levels [1,2,4,5,6]. Of the 23 qualifying trials on serum cholesterol with a garlic powder product, 43% found no effect [1]. Due to the inconsistencies, the trials have been the subject of several meta-analyses (nine on serum lipids and eight on blood pressure), with the overall conclusions being conservative [7]. Authors of the meta-analyses most frequently cite the high heterogeneity among the trials (high variation in dose, variable product types, identification of active compounds, standardization concerns, and unknown bioavailability) as the reason for caution in recommending garlic products for treatment of hypercholesterolemia and hypertension [1,4,5,7,8,9,10,11]. A recent review of the mostly in vitro antimicrobial effects of allicin concluded that determination of allicin bioavailability from various products is necessary before proper clinical studies can be conducted [12]. Hence, it is clear that attention needs to be given to the bioavailability and standardization of garlic’s active compounds under a variety of processing conditions, especially because of known or suspected potential issues with their formation, stability, metabolism, and detection in the body.

**Figure 1:**
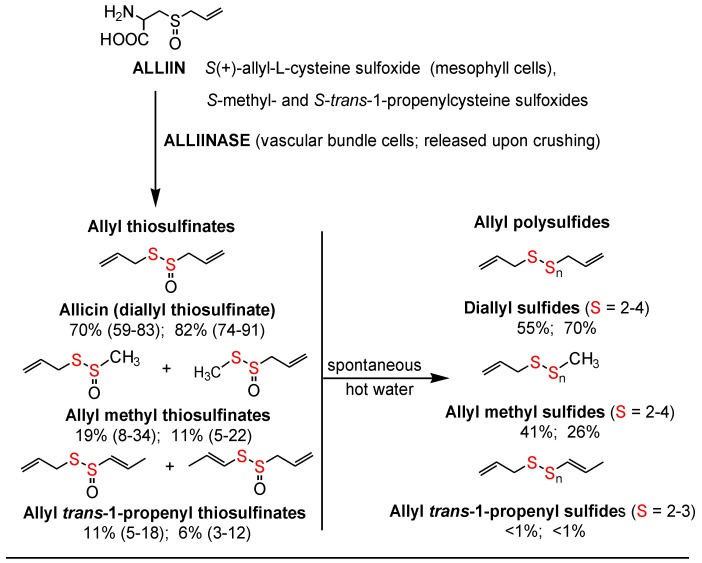

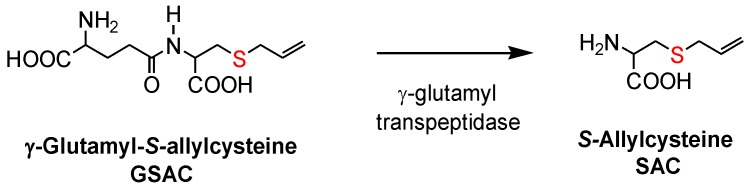
Structures of the known significant *S*-allyl compounds derived from garlic, including transformation reactions. The compounds above the horizontal line comprise alliin and alliin-derived dithioallyl compounds (AADD).The first set of values for the allyl thiosulfinates represent the average, minimum, and maximum mol % of total allyl thiosulfinates found for 26 samples from six countries; the second set is the *S*-allyl mol % or percent of the alliin transformed to each thiosulfinates—allicin increased because it is has two *S*-allyl groups [19,20]. The values for the allyl polysulfides represent the average mol % and *S*-allyl mol % for five samples of steam-distilled garlic oil capsules; small amounts of penta- and hexasulfides (3%) are also present in these oils [21,22] (p. 100). Spontaneous formation of the allyl polysulfides from the thiosulfinates (rapid in hot water, slow in ambient water) results in loss of about 25% of the total *S*-allyl, due to the formation of SO_2_, propene, and allyl alcohol [23,24].

**Figure 2:**
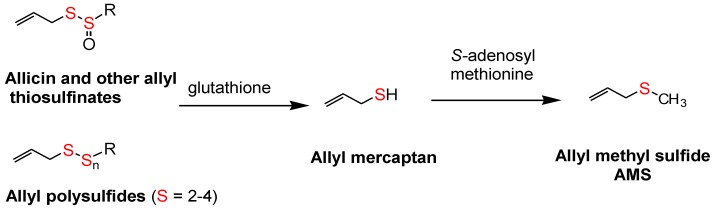
Metabolism of allyl thiosulfinates and allyl polysulfides. R represents allyl, methyl, or *trans*-1-propenyl. When R is allyl (allicin or diallyl sulfides), two moles of allyl mercaptan and allyl methyl sulfide (AMS) are formed.

The allyl thiosulfinates, of which allicin (diallyl thiosulfinate) is the most abundant and most studied member (Figure 1), are enzymatic products of alliin, *S*(+)-allyl-l-cysteine sulfoxide, and alliinase. They are rapidly formed when raw garlic cloves undergo cell rupture (Figure 1) or when dried and pulverized cloves (garlic powder) become wet [13,14]. The allyl thiosulfinates have been shown to be responsible for most of the pharmacological activity of crushed raw garlic cloves. Beginning in 1944 it was shown that allicin is responsible for the antibacterial activity of garlic and that selective removal of allicin also removed all activity [15,16]. Considerable evidence suggests that the allyl thiosulfinates, or their spontaneous transformation compounds (allyl polysulfides), or their common metabolite (allyl methyl sulfide, AMS), Figure 1 and Figure 2, are responsible for most of the lipid-lowering, antioxidant, anti-atherosclerotic, and anticancer effects of whole garlic, as observed in animals and humans [13,17]. Both allicin and γ-glutamyl-*S*-allylcysteine (GSAC), as the source of *S*-allylcysteine (SAC) (Figure 1), appear to be responsible for the hypotensive effects of garlic [18]. Indeed, no other compound has yet been shown to have significant activity at levels present in a normal human dose (3–5 g) of crushed raw garlic. The majority of the clinical trials on the possible cardiovascular effects of garlic have used supplements that are standardized on alliin or allicin potential [1,2].

Due to the abundance of alliinase [25], complete formation of allicin takes place in 0.5 min (5 min for the allyl methyl thiosulfinates) when water is added to garlic powder [14]. However, their formation in the body after consumption of garlic powder supplements is questionable because alliinase is inactive at pH 3.5 or below [14,26], a pH range commonly found in the stomach, although a moderate to high-protein meal can briefly raise the pH to 4.4 or higher [27,28], a range in which alliinase is active. Hence, many brands of garlic supplements have been enteric-coated to prevent disintegration in the stomach, and the U.S. Pharmacopeia (USP) has established a monograph for estimating allicin formation and release from such products under simulated gastrointestinal dissolution conditions [29]. However, 21 of 24 enteric brands subjected to this dissolution allicin release test yielded less than 20% of their allicin potential (the maximum yield of allicin from alliin upon activation of alliinase; the USP monograph refers to it as “potential allicin”), due to low tablet alliinase activity and to slow tablet disintegration [30]. Similarly, dissolution allicin release from non-enteric Kwai (Lichtwer Pharma, Berlin) tablets, the most commonly used garlic supplement in cardiovascular clinical trials [1,2], has also been found to be problematic: 44% for tablets made before 1993 and only 15% for tablets made from 1994–1997 [31].

The surprisingly low allicin release found under these standardized in vitro conditions indicates either that allicin release in the body from most garlic supplements is very low or that the U.S.P. dissolution test is not accurate. This dilemma highlights the need to determine the allicin bioavailability in vivo from garlic products, but attempts to do so have been troublesome. Allicin has been shown to be metabolized rapidly (half-life <1 min) to allyl mercaptan (allyl thiol) when added to whole blood [32], but neither allicin nor its transformation compounds (Figure 1) nor allyl mercaptan were found in the blood, urine or stool after volunteers consumed a large amount (25 g) of chopped raw garlic [33] (p. 152). However, it has been known for some time that AMS is a component of human breath after consuming raw garlic and that its rate of appearance and decline in the breath indicated it to be a product of systemic metabolism [34,35,36]. Lawson and Wang [17] conducted studies on human breath AMS and showed that (a) the area under the 32-h breath AMS concentration curve (AUC) is linearly proportional to the amount of allicin consumed; (b) AMS is the main breath metabolite of allicin, accounting for at least 90% of the allicin consumed; (c) allyl mercaptan is a temporary intermediate in the formation of AMS from allicin; (d) allicin-derived diallyl disulfide and diallyl trisulfide are also metabolized mainly to AMS; and (e) AMS is an active metabolite, responsible for the ability of allicin to increase breath acetone levels. By the use of a sulfur-selective detector, the sensitivity of the method was improved for the consumption of small amounts of garlic [37]. Hence, a validated method for determining the bioavailability of allicin from any garlic product has been established.

The term “allicin bioavailability” is being used to represent the sum of three processes: enzymatic (garlic alliinase) formation of allyl thiosulfinates (mainly allicin) from alliin, usually in the gastrointestinal tract, followed by their absorption and metabolism to a quantifiable metabolite, AMS. Allicin absorption is known to be highly efficient [38,39], although partial metabolism to the rapidly absorbed intermediate, allyl mercaptan, may occur during absorption [17]. The term “allicin bioequivalence” refers to the metabolic formation of the allicin metabolite, AMS, from any *S*-allyl compound, without the assistance of garlic alliinase, including allyl polysulfides, alliin and possibly other *S*-allyl compounds, such as GSAC and SAC; the term is being used in particular for products in which garlic alliinase is inactive. Together, the terms are referred to as allicin bioavailability or bioequivalence (ABB).

Although garlic supplements have been used in a large number of clinical trials, garlic is most commonly consumed as a food, usually as a cooked food and often as a commercial product suspended in an acid, such as whole cloves (pickled garlic) or small pieces (minced garlic), but no known clinical trials have been conducted with cooked or acidified garlic foods. However, the general public often wants to know if the results of clinical trials with supplements apply to garlic as a food, especially cooked garlic. In the U.S., cooked garlic is commonly prepared by boiling (soups) or roasting [40,41]. Because cooking or suspending garlic in acid fully inactivates alliinase [14,26], any allicin-related health benefits found with garlic supplements will probably be negligible in such foods. However, this logic assumes that the body itself—in the absence of active garlic alliinase—does not have the ability to metabolize alliin to allicin or allicin metabolites. Now that a method exists for determining the ABB of any garlic product, the validity of this assumption can be tested for alliinase-inhibited garlic foods.

The primary objective of this study has been to determine the problematic and hitherto unknown bioavailability of allicin from a variety of commonly consumed garlic supplements and garlic foods, in order to provide clinical trial researchers, manufacturers, and consumers with an improved knowledge basis when considering the possible health benefits of garlic products. The study has answered the following questions:

1. Can allicin bioavailability from garlic powder supplements be as high as that from crushed raw garlic?
2. Is the allicin bioavailability of the main garlic powder supplement (Kwai) used in clinical trials significantly less than that of raw garlic?
3. Is the allicin bioavailability of enteric garlic supplements greater than that of non-enteric ones?
4. Does the protein content of the meal being consumed with the supplement influence the allicin bioavailability?
5. How much supplement alliinase activity is necessary to achieve high allicin bioavailability?
6. What standards are recommended for the quality, content, and allicin bioavailability of garlic supplements being used in clinical trials?
7. Is the in vitro USP. dissolution allicin release test accurate for estimating allicin bioavailability from garlic supplements?
8. Do cooked garlic or acidified commercial garlic products have significant allicin bioequivalence? If so, how much of these foods would one need to consume to obtain the same ABB as raw garlic or a garlic powder supplement used in clinical trials?
9. Do garlic compounds other than alliin-derived compounds have significant allicin bioequivalence?

## 2. Materials and Methods

### 2.1. Standards

l(+)- and l(±)-*S*-allylcysteine sulfoxides (natural and racemic alliin), *S*-allylcysteine, diallyl disulfide, and diallyl trisulfide (each ≥98%) were purchased from LKT Laboratories, Inc. (St. Paul, MN, USA). Allyl methyl sulfide and diallyl sulfide (each 99%) were purchased from Sigma-Aldrich (St. Louis, MO, USA). γ-Glutamyl-*S*-allylcysteine (99%) was purchased from U.S. Pharmacopeia (Rockville, MD, USA). Allicin (98%) was prepared by oxidation of diallyl disulfide with hydrogen peroxide, followed by purification, as previously described [30]. Its concentration was validated with a second allicin standard prepared from alliin and crude alliinase [29]. Allyl methyl thiosulfinates, allyl-1-propenyl thiosulfinates, diallyl tetrasulfide, allyl methyl disulfide, allyl methyl trisulfide, and allyl methyl tetrasulfide were identified and quantified based on their relative retention times and relative extinction coefficients, compared to allicin and diallyl trisulfide, as previously described [19,21].

### 2.2. Control

The control for 100% ABB was prepared by homogenization of 500 g of peeled raw garlic cloves (California Late variety, Garlic World, Gilroy, CA, USA; 39.5% dry weight), after addition of water at 0.60 mL/g, in an Osterizer blender at the highest speed for 2 min. This allowed alliinase to transform all of the alliin to known amounts of allicin and other allyl thiosulfinates. The homogenate was divided into 50-mL jars and stored at −80 °C, under which condition the allyl thiosulfinates have been shown to be stable for at least 24 months [37]. Prior to consumption, the homogenate was thawed overnight at 4 °C, at which temperature the allyl thiosulfinates have been shown to be stable for at least 3 days [30]. After thawing, the homogenate was further macerated with a high speed Polytron homogenizer (Brinkmann, Kinematica AG, Luzern, Switzerland) with a 12-mm generator for 1 min at speed 6 of 10. This made it possible for the viscous homogenate to pass through the modified tip (drilled to 3 mm, inside diameter) of a 10-mL disposable syringe. At the time of consumption, a specific weight of the homogenate was transferred by syringe into the bottom of one or more size 0 hypromellose (hydroxypropyl methylcellulose) capsules (vegetarian capsules). The bottom of the size 0 capsule rested inside the bottom of a size 00 capsule to prevent aqueous allicin from coming in contact with the throat. The capsule was then closed with the top of a size 00 capsule. Under these conditions, the outer capsule remained firm and tasteless for 6 min. The standard control was 1.4 g of garlic homogenate, which was made from 0.88 g of raw garlic (0.35 g dry wt).

### 2.3. Supplements

All garlic supplements used in the study were purchased in 2009 at local stores or from online distributors and stored at ambient temperature. Only C1 was purchased directly from the manufacturer. All were tested by May 2011, within their claimed expiration dates (typically 2 years from the manufacture date, except 3 years for N1 and N4), with two intended exceptions (E6 and N4). Table 1 identifies the garlic supplements used in the study and includes the claims for garlic powder content (sometimes called garlic extract content or garlic dry weight), the actual tablet or capsule weights (average of at least 10 units), standardization claims, if any, and the recommended dose. All labels stated that the products should be consumed with a meal, except for N3. Table 2 gives the complete list of other ingredients for the supplements, with indications for the likely enteric coating agents when not specifically stated. The 13 supplements contained 51 different ingredients other than garlic powder.

### 2.4. Commercially Prepared Garlic Foods

Three common (purchased at grocery stores) and one less common (black garlic, purchased online) commercially prepared garlic foods were tested for ABB. The manufacturing procedures for all the products inactivates alliinase. Spicy Pickled Garlic (referred to as pickled garlic), manufactured by G.L. Mezzetta, Inc. (Napa Valley, CA, USA) consisted of whole raw cloves suspended in a medium of water, vinegar, crushed chili, and sodium bisulfite; cloves constituted 65% of the total volume. The pH of a 1:10 aqueous extract of the cloves was 4.3; the pH of the undiluted medium was also 4.3. Minced Garlic (referred to as acid-minced garlic), manufactured by Spice World, Inc. (Orlando, FL, USA) consisted of finely minced raw cloves mixed with a minimal amount of water (almost no standing liquid) and phosphoric acid; the pH of a 1:10 aqueous extract was 3.6. Chopped Garlic (referred to as oil-chopped garlic), manufactured by Christopher Ranch (Gilroy, CA, USA), consisted of finely chopped raw garlic mixed with soybean oil, olive oil (almost no standing liquid) and citric acid; the pH of a 1:10 aqueous extract was 4.3. The manufacturer’s web site calls it Chopped Garlic in Oil. Black garlic, manufactured in South Korea and distributed by Black Garlic, Inc. (Hayward, CA, USA), consisted of peeled whole cloves that were black (no additives).

### 2.5. Kitchen-Prepared Garlic Foods (Cooked and Raw Diced)

Roasted. Garlic (California Early variety, Garlic World, Gilroy, CA, USA) was roasted at two different temperatures commonly used for cooking (actual air temperature): 160 °C for 30 min and 215 °C for 60 min. Three garlic bulbs were placed on a rack inside a pre-heated Nesco 6 L electric roaster oven (Walmart). Wire probes from a calibrated Fluke 52II digital thermometer (Fluke Corp., Everett, WA, USA) were used to measure the temperature (recorded every 5–10 min) of the air inside the oven and the temperature inside the cloves of two different bulbs. The average temperature inside the cloves, after the first 10 min, was 92 °C for the 160 °C preparation and 101 °C for the 215 °C preparation. Roasting caused 5% and 25% loss of total weight, respectively. After cooling, the cloves were peeled and the soft contents thoroughly mashed and mixed. All of the contents of the 160 °C cloves were soft, but about half of the weight of the 215 °C cloves was hard and unusable. Roasting decreased the moisture content of the cloves from 63% to 58% and 55%, respectively.

Boiled. Unpeeled garlic cloves (California Early variety, Garlic World, Gilroy, CA, USA) were boiled for 4 min or for 45 min, by adding 24 cloves (80 g for 4 min or 110 g for 45 min) from six bulbs to 1.5 L of boiling water. The water resumed boiling about 15 s after the cloves were added. After boiling, the cloves were cooled, peeled, and thoroughly mashed and mixed. Preliminary studies showed that the temperature inside the cloves reached 95 °C (boiling point 96 °C) by 4 min if the clove weights were under 5 g or by 6 min if the clove weights were 5.5 to 7 g. It was also shown that alliinase activity was completely inhibited in 2 min for cloves weighing under 5 g. Hence, for the 4 min preparation, only cloves weighing 1.2 to 5.0 g were used, while cloves for the 45 min preparation weighed 1.2 to 9 g. The total weight of the cloves boiled for 4 min did not change, while the weight of the cloves boiled for 45 min increased by 5%. To reduce variation, cloves from each of the six bulbs were split into the two groups used for boiling.

Diced (cubed) raw. Raw garlic cloves (California Early variety, Garlic World, Gilroy, CA, USA) were peeled, then placed on a plastic grid of 3-mm lines, and cut with a razor blade into cubes measuring approximately 3 mm on each side (40 cubes per gram). This size is similar to that of commercial minced garlic. Two cloves from each of the same bulbs used for the boiling preparations were used. The cloves were carefully peeled and cut in order to minimize allicin formation.

### 2.6. Special Garlic Extracts

Protein-free, high-alliin garlic extract (PFHA). A garlic extract was prepared in which 97% of the soluble protein was removed, without loss of alliin. Garlic cloves (California Early variety, Garlic World, Gilroy, CA, USA) were peeled (100 g), boiled for 15 min in 1.5 L water (giving 14 mL water/g clove after evaporation loss), transferred to an Osterizer blender, homogenized at the highest speed for 1 min, filtered through cheesecloth, centrifuged at 1300× *g* for 20 min, subjected to 11 cycles of freezing at −22 °C and thawing, and finally centrifuged at 16,000× *g*. The soluble protein content decreased from 12.5 mg/g clove (before boiling) to 0.80 mg/g clove (before freeze-thawing) to 0.42 mg/g clove. The final clear extract contained 0.034 mg protein/mL.

Alliin-free, high-GSAC extract (AFHG). A garlic extract void of alliin and alliin-derived compounds was prepared to examine the effects of GSAC on breath AMS. Cloves (85 g), from the same 2-kg batch of bulbs that were used to prepare PFHA, were peeled, homogenized (2 min) in an Osterizer blender after addition of water at 14 mL/g (converted all of the alliin to allyl thiosulfinates), filtered through cheesecloth, boiled for 100 min, (causing all of the allyl thiosulfinates and resultant allyl sulfides to evaporate), and centrifuged at 1300× *g* for 20 min. Additional water was added as needed during boiling to make up for most of the evaporation loss. The soluble protein content decreased from 12.5 mg/g clove (before homogenization) to 2.4 mg/g clove (81% loss of protein). The final clear extract contained 0.25 mg protein/mL.

Alliin-free, high-SAC extract (AFHS). Product AFHG was treated with γ-glutamyl transpeptidase to convert 95% of the GSAC to SAC to learn if the two compounds have equal effects on breath AMS. A portion of AFHG (200 mL) was adjusted to pH 8.5 with 0.1 N NaOH, followed by the addition of 100 units of equine kidney γ-glutamyl transpeptidase (9.1 units/mg) (Sigma-Aldrich) and incubation at 37 °C for 3.5 h.

### 2.7. Participants

A total of 13 self-described healthy persons (6 female, 7 male) were recruited for the study in May–June 2009, all of whom continued throughout the 22 months of the study. Persons who had an intestinal disease, used tobacco, or frequently consumed alcohol, were excluded from the study.

The participant age range was 26–62 year (averages: total 35, men 34, women 36), with a body mass index (BMI) range of 22–35 kg/m^2^ (averages: total 26, men 28, women 25). The study was a series of bioassays; hence, it was not blind, nor was there a placebo or randomization Participants were always informed which garlic product they were consuming prior to consumption. Twenty-three types of garlic products were used in 43 tests, with usually 7–13 participants per test. A single set of bioavailability tests required two days to conduct; they were usually conducted two times per week and included 3–4 participants per set. Participants usually participated in two tests per month and were paid for each test. A record of all interactions with the subjects during a test, along with any report of side effects, was maintained on set consumption records. All bioavailability tests with garlic products and garlic extracts, along with the consent form and research protocol, were approved by the Western Institutional Review Board, Olympia, Washington, USA, on 18 March 2009 (study number 1105434).

### 2.8. Standard Breakfast Meals and Garlic Product Consumption

All garlic supplements, the homogenized raw garlic (control), and the extracts, were consumed with either a standardized low-protein meal (LP) or a standardized high-protein meal (HP). The LP meal consisted of two slices of toasted white bread, 19 g of butter, 30 g of jam, a banana, and 200 mL of water; it contained 5.5 g of protein and 17 g of fat. The HP meal consisted of a whole wheat tuna sandwich and 200 mL of whole milk, including two slices of bread, 54 g of pressed canned albacore solid white tuna, and 34 g of garlic-free light mayonnaise; it contained 31 g of protein and 19 g of fat. The meals were isocaloric (460 kcal). The gastric pH is lowest before a meal (av 1.9) and quickly rises (av 14 min), depending upon the meal protein content, to a maximum pH of 4.4–6.7 after starting a meal [28,42]. Hence, the supplements were consumed immediately before the LP meal, to allow exposure to approximately the lowest gastric pH attainable when they are consumed with a meal—or immediately after the HP meal, to allow exposure to a substantially higher gastric pH. The control, in capsules, was always consumed immediately after the LP or HP meals to reduce the chance of stomach disturbance. For the control, gastric pH was not a concern, as alliinase had been activated before consumption. Garlic foods (cooked garlic, etc.) and their control were consumed inside the LP meal, with the garlic placed between the two slices of buttered bread and the jam omitted. The special extracts (clear aqueous liquids) were consumed with the LP meal.

### 2.9. Diet Restrictions

It was essential to prevent dietary interference with the bioavailability test by restricting the consumption of garlic and onions for two days before each test and for the 1.5 days of each test. The restrictions were as follows: two days before a test, only modest amounts of garlic; one day before the test and during the test, no foods containing garlic or onion, with some exceptions. Some prepared foods listing garlic were found to contain too little garlic to cause breath AMS production: Kraft Miracle Whip, Pace Chunky Salsa, and Doritos Cool Ranch Chips. A small amount of cooked onion caused no interference. Prepared foods, such as ketchup, mustard, salad dressing, tomato sauce, and chips, which listed onion as a spice, but not garlic, caused no interference. Participants were discouraged from eating restaurant food unless they were sure garlic and onion were absent from their choices.

On the day of each test, participants were asked to not eat or drink until arriving at the facility, except, if necessary, up to 150 mL water one hour before arrival. After consuming the standard breakfast meal and garlic product, participants were asked to not eat or drink again until two hours later. However, if they felt discomfort during the two hours, small amounts of non-protein food were permitted. Participants were occasionally asked if they had adhered to these restrictions.

Compliance with the diet restrictions was determined by the absence of AMS and the presence of no more than a modest amount of onion sulfides in a breath sample taken immediately before the test product was consumed. Non-compliance after the test began was monitored by a sudden increase in breath AMS concentration at 2–3 h after consumption of the subsequent meals consumed during the test. If non-compliance was determined, participants were asked to stop the test and repeat it at a later date.

### 2.10. Breath Sampling Procedure

Participants breathed into 1.2-L Tedlar^®^ gas sampling bags (Grace Davison Discovery Sciences, Waukegan, IL, USA), fitted with a septum port, a barbed nickel-plated twist-valve, and a 4-cm length of Tygon^®^ tubing, as a mouthpiece, attached to the valve. They were instructed to exhale a normal breath, starting at the top of the breath (avoiding the natural tendency to first take a deep breath), into the bag, then to empty the bag by flattening it, followed by exhaling a second breath until the bag was at least mostly full and closing the valve. After the flattening, the mouthpiece was placed against the tongue or lip to prevent air from entering the bag until the participant was ready to exhale the second breath. The speed at which one breathes into the bag was found to have no influence on the breath AMS concentration. Participants recorded their name and breath time on each bag.

Whole breath, rather than alveolar breath, was used throughout the study. Although alveolar breath has been shown to contain 18% higher AMS than whole breath [17], the procedure for sampling alveolar breath was considered too difficult for participants to use away from the laboratory, where most of the 17 breaths per test needed to be collected. Because all tests were conducted in the same manner and because only relative amounts are important, using whole breath was considered sufficient.

### 2.11. Breath Bag Quality

The average rate of disappearance of AMS from the Tedlar^®^ bags was 0.4%/h (range 0.05–1.05%/h). The disappearance rate for each bag was determined over a 48-h period and only the better bags (<0.50% loss/h) were used for the breaths that needed to be stored overnight before analysis. Except for the overnight breaths, breaths were usually analyzed within 4–6 h after sampling. Following these guidelines, losses were considered insignificant and not corrected. Moisture accumulation in the bags was found to have no effect on the AMS concentration.

### 2.12. Test Protocol

After an overnight fast, participants came to the facility, breathed into a bag and then consumed the standardized breakfast meal and the garlic product. After consuming the garlic product, participants left the facility with several empty bags and provided breath samples every hour for the next eight hours, then every two hours, except during sleep, until breath AMS was undetectable (typically 24–30 h), but not longer than 32 h. Participants returned to the facility to deliver breath samples and obtain more bags at about noon and 5 pm of the first day and at about 8 am and 2 pm of the second day.

### 2.13. Breath Analysis for Allyl Methyl Sulfide (AMS)

Breath samples (5 mL) were injected directly (manually with a 5-mL gas-tight syringe fitted with a 0.63 mm side-port needle), one time, into a gas chromatograph (Agilent 6890) fitted with a model 5380 sulfur-selective pulsed flame photometric detector (PFPD, OI Analytical, College Station, TX, USA), PFPD zero-output setting of 10, and a 30 m × 0.32 mm × 4 μm SPB-1 Sulfur (bonded polydimethylpolysiloxane) capillary column (Sigma-Aldrich #24158). Helium was the carrier gas (1.6 mL/min, constant flow). The column temperature was programmed from 45 °C (0.2 min) to 200 °C (1.2 min) at 50°/min, with a hold time of 2 min, giving a retention time of about 4.0 min, a run time of 5 min, and a re-injection time of 11 min. The injection port contained a straight 4 mm borosilicate liner (Agilent #19251-60540) and was operated at 175 °C in the splitless mode, with a purge flow of 45 mL/min for 0.8 min. The detector was operated at 250 °C, with the following flow rates: hydrogen 9.5 mL/min, air 18.5 mL/min, helium (make-up) 5.5 mL/min. Due to injection of an overload volume of 5 mL and a rapid temperature gradient, the baseline did not become stable until 2.9–3.0 min after each injection. Because inserting the column into the detector requires disassembly of the detector and several hours of equilibration time, a 40-cm piece of the column (extension) was attached to the detector, with the other end attached to an Agilent Ultimate Union (G3182-61580). The remainder of the column was then attached to the extension at the union. This allowed for quick removal and replacement of the column for other uses of the gas chromatograph. The PFPD peak area is the square root of the detector response to sulfur. This detector gave about 20 times greater sensitivity (detection limit, 40 area units or 3 ng/L or 1 ppb at s/n = 2) than the FID (flame ionization detector) detector used in prior allicin bioavailability studies [17], which made it possible to measure the AUC after consuming small amounts of garlic products. AMS is normally absent from human breath [43].

### 2.14. AMS Standard Curve

**Figure 3:**
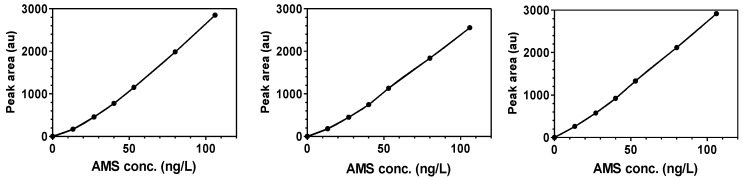
Examples of standard curves for AMS standards, 13–106 ng/L, over the course of 14 months.

The stock allyl methyl sulfide vapor standard (266 ng/L) was prepared by adding 13.0 μL of a solution of 88 μg allyl methyl sulfide/mL in methanol to duplicate 4.3-L glass jugs with lids fitted with septum ports. The concentration in the jugs reached stability by 2–3 h and remained stable for 24–30 h. Dilutions of this standard gave a curvilinear linear response down to 6 ng/L. Dilutions were prepared by adding various volumes of the stock (0.25–3 mL) and air (4.75–2 mL) to a 5-mL syringe and injecting the 5-mL. For volumes less than 1 mL, the stock was added to the 5-mL syringe using a 1-mL syringe. An air-blank was injected daily. When eight standard curves, created over a 14-month period (see examples in Figure 3), were applied to a single set of breath data, the resultant AUC was found to vary by only 5% (RSD, relative standard deviation), indicating the stability of the PFPD detector response.

### 2.15. AMS Concentration Curve (AUC)

The AUC was measured in ng-h/L by determining the AMS concentration of each breath, based on AMS peak areas and interpolated concentration values (Spline Lowess regression) from the standard curve, followed by determining the AUC over 32 h. All of the calculations were determined using GraphPad Prism 5.0 (San Diego, CA, USA) software.

### 2.16. Allicin Bioavailability or Bioequivalence

The ABB for each product was based on response factors (RF, RF2, RF3) and calculated as relative response factors (RRF, RRF2, RRF3). RF is the ratio of AUC to the μmol of alliin and alliin-derived dithioallyl compounds (AADD, see Figure 1) consumed. RF2 is the ratio of AUC to the μmol of total known *S*-allyl compounds (TKSA) consumed; TKSA is AADD plus GSAC and SAC (G/SAC). RF2 was especially useful when AADD was very low and G/SAC were dominant. RF3 is the ratio of AUC to the weight (g) of product consumed; it was used mainly for “aged” garlic products, such as black garlic, in which most of the alliin has been converted to unidentified *S*-allyl compounds. The relative response factors were calculated as 100% times the response factors for the products divided by the response factors for the standard control. When products contained alliinase activity (the supplements and diced raw garlic), allicin bioavailability was determined based on AADD. When the alliinase of garlic products had been inhibited (cooked, acidified), allicin bioequivalence was determined based either on AADD or TKSA. The minimum AUC that could be accurately determined was 15 ng-h/L, when there was a relatively sharp decline in AMS after Tmax was reached (typical when the product had active alliinase, such as with the control and N1). It was about 45 ng-h/L when there was a gradual decline after the Tmax (typical when alliinase was absent, such as with PHFA, AFHG, and alliin). When the product contained active alliinase, ABB as low as 0.7–11% could be determined when the AADD consumed was 320–20 μmol, respectively. When the product did not contain active alliinase, ABB as low as 2–32% could be determined when the AADD consumed was 320–20 μmol.

### 2.17. Dissolution Allicin Release

The formation and release of allicin from garlic tablets and capsules under simulated gastrointestinal conditions was determined according to the USP-NF (U.S. Pharmacopeia-National Formulary) dissolution method for delayed-release garlic tablets [29]. Using a model VK 700 dissolution apparatus (Agilent/Varian, Palo Alto, CA, USA) equilibrated at 37 °C, 2–6 tablets or capsules (enough to provide 20–30 mg alliin) were placed into each of two covered 1-L round bottom glass vessels containing 750 mL of 0.1 N HCl and paddle-stirred at 100 rpm for 2 h, after which 250 mL of 0.2 M Na_3_PO_4_ was added and the pH slightly adjusted, if necessary, giving 1000 mL at pH 6.80 ± 0.05. After stirring the buffered medium for 60 min, 1 mL was added to 0.05 mL of 210 mM (final 10 mM) carboxymethoxylamine (CMA) (Sigma-Aldrich) alliinase inhibitor, followed by high-performance liquid chromatography (HPLC) analysis of allicin. For tablets that had not completely disintegrated in 60 min of buffer, an additional 1 mL aliquot was taken upon complete tablet disintegration. Capsules were loosely wrapped with three winds of plastic-covered wire to keep them from floating. The time to achieve complete disintegration was determined by observation during the dissolution test. The percent dissolution allicin release (DAR) was calculated as the amount of allicin released during the dissolution tests divided by the amount of allicin released when pulverized tablets or capsule contents were incubated 30 min in water to allow complete allicin formation (allicin potential).

### 2.18. Analysis of Garlic Products 

Garlic supplements (tablets and capsule contents) were mortar ground, extracted with 20 mM CMA alliinase inhibitor (50–100 mL/g) for analysis of alliin, GSAC, and SAC, or extracted with 70% acetonitrile (ACN)/30% 40 mM CMA (10 mL/g) for analysis of allyl sulfides. For water activation of alliinase to generate the allyl thiosulfinates, ground tablets or the contents of capsules were mixed with water at 50 mL/g, rotated rapidly for 10 min, centrifuged for 5 min at 1000× *g*, followed by addition of one volume of ACN (precipitates protein), centrifuged for 3 min at 16,000× *g*, and kept at 4 °C or lower until analyzed [37]. Commercial garlic foods were extracted with a Polytron homogenizer at speed 6 for 15 s, in the presence of either 20 mM CMA (10 mL/g) for analysis of alliin, GSAC, and SAC or in the presence of 75% ACN/25% water (10 mL/g) for analysis of allyl thiosulfinates and allyl sulfides. The control (raw garlic homogenate) was extracted with 20 mM CMA (20 mL/g) for analysis of alliin, GSAC, and SAC, or with 50% ACN (10 mL/g) for analysis of allyl thiosulfinates, or with 70% ACN (10 mL/g) for analysis of allyl sulfides. Diced raw garlic was extracted with a Polytron homogenizer (speed 6, 15 s) in the presence of 20 mM CMA (25 mL/g) for analysis of alliin, GSAC, SAC, and, after addition of 1 volume of ACN, for analysis of allyl thiosulfinates and allyl sulfides. Roasted or boiled garlic were extracted with a Polytron homogenizer (speed 6, 15 s) in the presence of water (25 mL/g) for analysis of alliin, GSAC, and SAC, or in the presence of 75% ACN (10 mL/g) for analysis of allyl thiosulfinates and allyl sulfides.

Alliin, GSAC, and SAC were analyzed by ion-pair HPLC (250 mm × 4.6 mm, Agilent/Varian Microsorb-MV 100-5 C18 250 mm × 4.6 mm column) at 208 nm by modification of the method of Arnault et al. [44]. Solvent A consisted of 20 mM sodium heptanesulfonate (ion-pair agent) (Sigma-Aldrich) and 20 mM sodium phosphate monobasic, pH adjusted to 2.1 with concentrated phosphoric acid. Solvent B was ACN. The column was heated at 38 °C with a flow rate of 1.0 mL/min. Gradient: from 0% B to 15% B in 5 min, then to 22% B by 15 min and hold until 17 min, then back to 0% B by 18 min and hold until 24 min. Typical retention times: l(−)-alliin 8.5 min, l(+)-alliin 8.8 min, isoalliin 9.1 min, γ-glutamyl-*S*-methylcysteine 11.0 min, *S*-allylcysteine 13.7 min, γ-glutamyl-*S*-allylcysteine 15.9 min, γ-glutamyl-*S*-*cis*-1-propenylcysteine 17.3 min, γ-glutamyl-*S-trans*-1-propenylcysteine 17.5 min, γ-glutamylphenylalanine 18.5 min. The allyl sulfides and allyl thiosulfinates (when their abundance was small) were analyzed, using the same column, isocratically with 70% ACN/30% water at 210 nm, as previously described [21] (1991 sulfides pub). When the allyl thiosulfinates were abundant, such as with the control, or when the dissolution allicin release and USP potential allicin (allicin potential) were determined, they were analyzed isocratically with 45% ACN/55% water at 240 nm, as previously described [37].

### 2.19. Alliinase Assay

Alliinase activity, as μg allicin produced min^−1^ g^−1^ garlic powder (dry garlic matter), was determined as previously described [30]. Briefly, the capsule contents or mortar-pulverized tablets, without sieving, were added to water at a concentration of 1 g garlic powder content to 800 mL water, followed by immediate and vigorous shaking for 7–8 s and removal of 1 mL aliquots at 15, 30, 60, and 120 s to 0.05 mL of 210 mM CMA to stop the reaction. After analysis of allicin, the alliinase activity was determined from the time point giving the highest rate of allicin production. If the activity was found to be less than 500, it was redetermined at 200 mL/g garlic powder. The garlic powder content for tablets and capsules was based on label claims.

### 2.20. Protein Assay

The protein content of extracts PFHA and AFHG were assayed according to the procedure of Bradford [45], using Brilliant Blue G (Sigma-Aldrich) in methanol as the binding dye and bovine serum albumin (Sigma-Aldrich) as the protein standard (standard curve 10–100 μg/mL).

### 2.21. Peptide Hydrolysis

To reveal any possible peptide-bound alliin or SAC, both AFHG extract and roasted garlic prepared at 160 °C (homogenized with 10 mL water/g) were incubated while stirring at pH 8.5 at 37 °C for one hour with a large excess of either protease from *Bacillus licheniformis* (9.6 units/mg, Sigma-Aldrich) or alkaline protease from *Bacillus subtilis* (400 PC units/mg, Bio-Cat, Inc., Troy, VA, USA), followed by one hour of incubation with porcine kidney γ-glutamyl transpeptidase (3.4 units/mg, Sigma-Aldrich). Aliquots were centrifuged at 16,000× *g* and directly assayed for alliin, SAC, and GSAC as described in Section 2.18.

### 2.22. Statistical Analysis

Statistical analyses were conducted using Microsoft Excel Analysis ToolPak software (Redmond, WA, USA). Student’s *t*-Test (paired two sample for means; two-tail) was used to determine differences between groups. In a few cases, the two-sample *t*-Test was used when *n* was small (≤4). *p*-Values < 0.05 were considered to be significant. Data are presented as means ± standard deviation (SD).

## 3. Results

### 3.1. AMS Peak Resolution and Interference from Onion Sulfides

AMS (retention time 4.0 min) was well-resolved from other volatile sulfur compounds derived from garlic: allyl mercaptan (3.3 min), diallyl sulfide (4.9 min), and diallyl disulfide (6.9 min), although none of these compounds were detected when the breath was sampled at ≥0.5 h after garlic product consumption. AMS was almost baseline separated from XMS (4.2 min), the only volatile sulfur compound detected after consuming 5 g cooked onion. Upon consuming raw onion, only two peaks were found: XMS and YMS (4.1 min). AMS was not well-separated from YMS; hence, it was important to restrict study participants from eating raw onion. Consumption of small amounts of cooked onion did not cause interference. No sulfur compounds were detected in the breath unless garlic or onion had been consumed.

The identities of XMS and YMS were not confirmed by gas chromatography–mass spectrometry (GC–MS); however, XMS is probably *trans*-1-propenyl methyl sulfide, which is often found in human breath [43], and YMS co-eluted with pure n-propyl methyl sulfide (Alfa Aesar, Tewksbury, MA, USA). For cooked onion, these sulfides would be metabolites of isoalliin (*S*-*trans*-1-propenylcysteine sulfoxide), the main sulfur compound found in onion, and propiin (*S*-n-propylcysteine sulfoxide), which is only 3–10% as abundant [46,47]. For raw onion, they would be the metabolites of the respective 1-propenyl and propyl thiosulfinates. In contrast to the much greater abundance of isoalliin than propiin in whole raw onion, consumption of raw onion resulted in 6-fold more YMS than XMS. This probably occurred because, after the action of alliinase and lachrymatory factor synthetase upon crushing raw onion, isoalliin is mainly (80–90%) transformed to highly volatile lachrymatory factor (propanethial *S*-oxide, CH_3_CH_2_CH=*S*-O) and only a small amount (5–10%) to 1-propenyl thiosulfinates, while propiin is transformed only to propyl thiosulfinates [24,46,47]. It is also possible that 1-propenyl thiosulfinates are metabolized to the methyl sulfides less efficiently than are the propyl thiosulfinates. A small amount of XMS was often seen after consuming garlic products, probably due to the presence of a small amount isoalliin in garlic and the subsequent 1-propenyl thiosulfinates found in crushed raw garlic [19,48].

### 3.2. Example Breath AMS Concentration Curves

**Figure 4:**
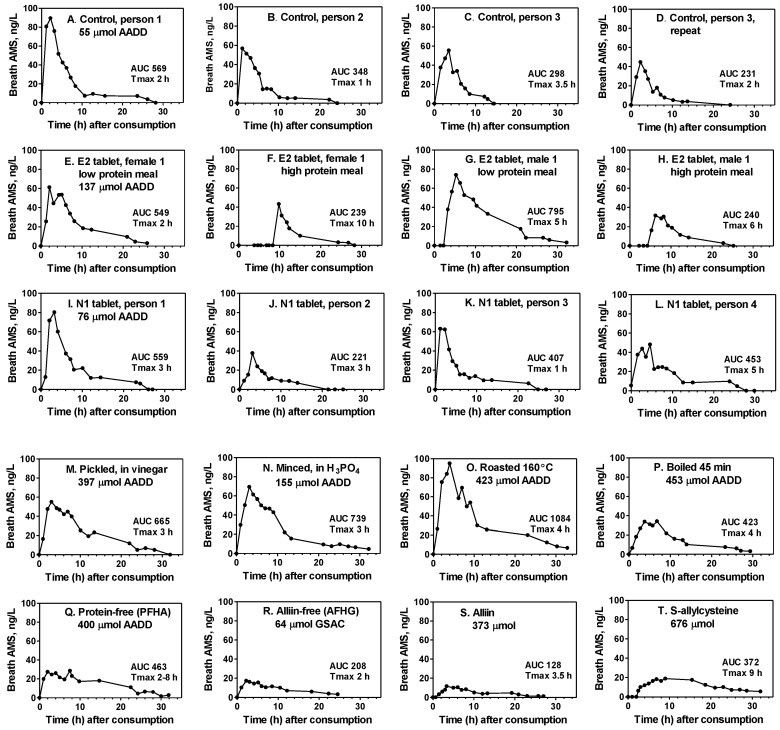
Typical concentration curves for breath AMS, after consumption of various garlic products. (**A**–**D**), 1.4 g of raw garlic homogenate (0.88 g raw garlic) consumed in capsules with low-protein meal (the standard control). (**E**–**H**), enteric-coated tablets, brand E2 (2 tablets), consumed with low or high-protein meals by a female or male. (**I**–**L**), normal tablets, brand N1 (3 tablets), consumed with low-protein meal (not the same persons as A–D). (**M**–**P**), garlic foods consumed with low-protein meals (four different persons): **M** = 12 g pickled garlic; **N** = 7 g acid-minced garlic; **O** = 6.1 g roasted garlic (160 °C for 30 min); **P** = 5.5 g boiled garlic (45 min). (**Q**–**T**), garlic extracts and pure compounds: **Q** = protein-free high alliin extract (PFHA); **R** = alliin-free high GSAC extract (AFHG); **S** = pure l(+)-alliin; **T** = pure *S*-allylcysteine. S and T were consumed by the same person. Q and R were consumed by another person, but very similar curves were found when Q and R were consumed by the person who consumed S and T.

Typical breath AMS concentration curves for several of the products consumed in the study are illustrated in Figure 4. They depict hourly fluctuations, the Tmax (time to achieve maximum breath concentration), the AUC relative to the amount consumed (AADD), and delays in AMS production (F, G, H, T). They also demonstrate the variation between persons for the standard control (A–C) and N1 tablets (I–L), the variation between duplicate tests by the same person (C and D), and the effect of a high meal protein content on reduced AUC and delayed AMS production when enteric-coated tablets are consumed (E–H). The substantial production of breath AMS, even when active garlic alliinase is not present, is shown for both acid-processed and cooked garlic foods (M–P) as well as for extracts and pure *S*-allyl compounds (Q–T).

### 3.3. Dose Response

**Figure 5:**
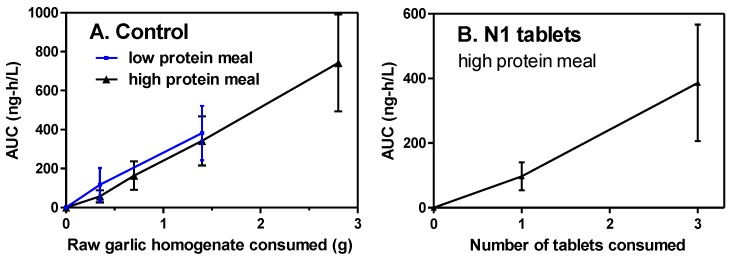
Dose response of the breath AMS concentration curve (AUC) after consumption of A, the control (raw garlic homogenate) or B, N1 tablets, with high- or low-protein meals. Values are means ± standard deviation (SD) (*n* = 13).

The response of the breath AMS-AUC to consumption of 0.35, 0.70, 1.4, and 2.8 g of the raw homogenate control (0.22–1.76 g raw garlic) under high-protein meal conditions was determined for all 13 participants (Figure 5). The allicin content of the control was 0.87–6.94 mg per dose and was fully present before consumption. The response was dose-dependent (r^2^ = 0.9989) and gave high linearity through 0.35 g. Under low-protein meal conditions, only two doses were used, 0.35 and 1.4 g, which closely paralleled the result with the high-protein meal. A dose of 1.4 g with the low-protein meal was chosen as the standard control condition. N1 tablets, where allicin was absent until made in the body, were tested at two doses with a high-protein meal. The response was dose-dependent (r^2^ = 0.9975) and moderately linear.

### 3.4. Experimental Variation

The experimental variation is summarized in Table 3. The variation (RSD) in the AMS peak area between triplicate injections of the same breath was 3.4% (*n* = 12), with no significant difference (*p* > 0.4) between breaths containing a high AMS concentration (88 ng/L, peak area 2110 au), a moderate concentration (42 ng/L, peak area 860 au), or a low concentration (10 ng/L, peak area 180 au). The variation between triplicate breaths taken 1 min apart was a little greater, 5.6%, as determined for four persons, and also includes the variation between replicate injections of the same breath. The average variation in the AUC between all 13 persons consuming the same product was 42 ± 7.5% (range 32–63%) for 21 tests with garlic supplements and the control. However, this variation decreased to 32 ± 9.9%, a significant (*p <* 0.001) 24% decrease, by comparing the AUC for a product to the control AUC value found for each person, the relative response (AUC/AUC_control_). The variation was independent of meal type and supplement type.

The variation in the AUC for duplicate consumption of a garlic product by the same person was determined three times for all 13 participants, which revealed an overall average within-person variation of 16.9%. When 1.4 g of the raw homogenate control was consumed with a low-protein meal, the variation was 14.4 ± 11.5%. When 1.4 g of the raw homogenate control was consumed with a high-protein meal, the variation was 17.4 ± 15.4%. When three N1 tablets were consumed with a high-protein meal, the duplicate variation was 18.7 ± 14.0%. The variation in the average AUC for duplicate sets of tests with the same product, same dose, and same meal type for the 13 persons was 4.7%. Duplicate test sets were conducted three times: 1.4 g of control with the low-protein meal (1.2% RSD), 1.4 g of control with the high-protein meal (3.9% RSD), and three N1 tablets with the high-protein meal (9.0% RSD).

### 3.5. Composition

The TKSA content of the garlic products consumed in the study is described in Table 4. Alliin was absent from the homogenized raw garlic used as the control because it had been converted to allicin and other allyl thiosulfinates during homogenization; however, the original alliin content of the cloves—calculated from the allyl thiosulfinates produced—was 154 μmol/g dry wt (60.9 μmol/g fr wt) or 27.3 mg/g dry wt (10.8 mg/g fr wt), which values are close to the average values reported for 46 samples: 156 ± 33 μmol/g dry wt (54.7 μmol/g fr wt) or 27.7 ± 5.8 mg/g dry wt (9.7 ± 2.0 mg/g fr wt) [49,50,51]. Among the garlic supplements and foods, alliin was usually the most abundant *S*-allyl compound, typically accounting for 65 to 85% of the total, but it was lower, 30–40%, for products E5, N2, and oil-chopped garlic, while being undetectable in black garlic. The alliin concentration in the garlic powders used in the supplements varied 7.5-fold (6.5–49; 21.0 ± 12.2 mg/g) (Table 4, second set of alliin values). This range is substantially broader than the 2-fold range consistently found for garlic bulbs among sets of 13, 9, and 24 cultivars [49,50,51]; this indicates variable loss of alliin upon manufacture of garlic powders and validates the need for the allicin potential or alliin standardization label claims found for most brands (Table 1).

Alliin consists of both the l(+)-isomer and the l(−)-isomer. The l(−)-isomer, which tends to increase with age, constituted <6% of the alliin for most of the products, the exceptions being E4 (8%), E5 (14%), E6 (24%), N1 (8%), N2 (10%), N4 (19%), C2 (9%), C3 (15%), pickled (11%), acid-minced (18%), and oil-chopped (24%). Second in abundance was GSAC, which accounted for 8–35% of the total *S*-allyl, with E5 and N2 being exceptionally high (57–58%). SAC accounted for 2–6% of the total, except 13% for C3 and 86% for black garlic. Of the total SAC (GSAC + SAC), SAC typically accounted for 1–15%, exceptions being E3 (36%), pickled garlic (44%), and black garlic (87%).

As intended, alliin, allyl thiosulfinates and allyl polysulfides were absent from the AFHG and AFHS extracts, and treatment of AFHG with γ-glutamyl transpeptidase to produce AFHS converted 95% of the GSAC to SAC. Allicin and other allyl thiosulfinates were absent from all supplements because alliinase had not been activated. Upon activation in water (Section 2.18), the following average yields and ranges were found (*n* = 13, not shown in Table 4): (a) as mg/g garlic powder (powder weight claims are in Table 1)—allicin 7.8 ± 4.8 (2.4–16.8), allyl methyl thiosulfinates 1.7 ± 1.1 (0.5–4.6), allyl *trans*-1-propenyl thiosulfinates 0.3 ± 0.4 (0.01–1.4); (b) as mg/tablet or capsule—allicin 3.6 ± 2.9 (0.3–10.8), allyl methyl thiosulfinates 0.8 ± 0.6 (0.05–2.1), allyl *trans*-1-propenyl thiosulfinates 0.2 ± 0.2 (0.002–0.6). The allyl thiosulfinates were absent from homogenized commercial garlic foods because alliinase had been inhibited by the processing acids (vinegar, phosphoric acid, citric acid). They were abundant in the homogenized control, where allicin accounted for 80.3 *S*-allyl mol % or 70.3 wt % of the allyl thiosulfinates, and they were slightly present in the roasted, boiled, and diced raw garlic preparations. Dicing cloves into 3 mm cubes caused only about 3% of the alliin to be transformed to thiosulfinates, while homogenization in water caused complete transformation.

The allyl polysulfides, which are metabolized to AMS to the same extent as allicin [17], were significant (>5% of the AADD) only in E5 (14%), acid-minced garlic (14%), and oil-chopped garlic (51%). Oil-chopped garlic uniquely contained cyclic vinyldithiins, 2-vinyl-4*H*-1,3-dithiin and 3-vinyl-4*H*-1,2-dithiin (5.4 μmol/2.5 g), which are not metabolized to AMS, and a small amount of ajoene (0.1 μmol/2.5 g), which is metabolized to AMS [17]. Of these products, the diallyl sulfides comprised 82–94% of the total allyl sulfides, with diallyl trisulfide always dominant, as detailed in the footnotes of Table 4. AMS itself was not found in any product except in oil-chopped garlic (0.7% of the AADD).

### 3.6. Control: Allicin Bioavailability or Bioequivalence

The response factor (RF) for 1.4 g of the control was used for determining the ABB of all other products. Hence, it was important to establish its variation between duplicate tests, its variation with respect to meal types, its variation with the mode of consumption, and its variation over the duration of the study. The results for the individual duplicate tests and their averages for consumption with the high-protein or low-protein meals are shown in Table 5. The variation in the AUC and RF between duplicate tests was low for both the high-protein meal (RSD 3.9%) and the low-protein meal (RSD 1.2%). Compared to the low-protein meal, consuming the control with a high-protein meal significantly decreased (*p* = 0.023) the RF by 18% (7.37 vs. 6.25), delayed the Tmax by 0.7 h and decreased the Cmax by 25%. Apparently, some of the allicin binds to or reacts with the milk protein or tuna protein or other components in the high-protein meal without forming a reaction product that can be transformed to AMS. The RF was not significantly different between men and women for either the low-protein tests (*p* = 0.56) or the high-protein tests (*p* = 0.78).

The control was also consumed in the same two modes in which the various garlic products were consumed. Similar to the consumption of supplements, it was consumed in capsules, giving the gastrointestinal tract initial contact with allicin and other garlic compounds. Garlic foods were consumed inside a low-protein sandwich, giving the mouth initial contact with the garlic compounds; hence, the control was also consumed inside the same sandwich. The RF for the control consumed inside the sandwich (Table 5, test 3) was not found to be significantly different (*p* = 0.48) than consumption in capsules (7.31 vs. 6.99). As expected, consumption in a bread sandwich significantly decreased the Tmax from 2.6 h to 1.4 h and doubled the breath AMS concentration at 1 h after consumption (% of Cmax at 1 h, an evaluation of Tmin).

It was critical to the validity of the bioavailability control to consume it under conditions that allowed for maximum allicin availability (maximum RF). Based on the factors presented in Table 5, the standard control RF used was the value found upon consumption of the control in capsules with the low-protein meal (test 2). Tests 2a and 2b were conducted just prior to the tests with the supplements. Because of the long duration needed to conduct most of the supplement tests, 10 months, an additional standard control test (test 2c) was conducted 9 months after the initial tests. The average RF for all three standard tests (test 2, final = 6.99 ± 2.39) was used for determining ABB for all the garlic products, regardless of the type of meal with which they were consumed.

### 3.7. Garlic Supplements: Allicin Bioavailability

#### 3.7.1. Dose Basis

The dose used for the supplement bioavailability tests was not based on manufacturer recommendations (Table 1), which varied from 1–6 units per day (garlic powder range of 0.3 to 2.0 g, average 1.0) or from 1–3 units per meal (garlic powder range of 0.1–1.5 g, average 0.6 g). Rather, it was based on the amount needed to give an AUC value within or near the tested linear range that was found for the control (100–750 ng-h/L, Figure 5a), as determined from a preliminary test for each product with a person who gave a typical response. It was also desired to consume at least two units to obtain a better average response. In one case, N3, only 1 tablet was consumed because of its very large size (1.8 g) and high AUC. If there was low alliinase activity, a higher dose was used. Typically, the dose consumed was 1–2 times the recommended daily dose or 2–3 times the recommended dose per meal.

#### 3.7.2. Enteric-Coated Tablets

Under low-protein meal conditions, allicin bioavailability from brands E1, E4, and E5 was not significantly different from that of the 100% bioavailability control, but the bioavailability for brands E2 and E3 was significantly lower, especially for E3 (Table 6). Four of the five brands gave medium to high allicin bioavailability (>65%), while E3 gave moderate bioavailability (35–65%). Even brand E6, which was 12 years old, also gave moderate bioavailability. The delay in tablet disintegration and allicin formation caused by the enteric coatings is indicated by both the Tmax and the Tmin (the first measured time for AMS to appear in the breath), relative to the garlic homogenate control that contained allicin before consumption. However, because the first breath was not sampled until one hour after consumption, the true Tmin is not known for any garlic product with a Tmin of less than 1 h. The true Tmin for the control was determined for one person by taking breath samples every 6–8 min for the first hour and was found to be 26 min if consumed immediately after the low-protein meal or 15 min if consumed immediately before the same meal. As learned from the % of Cmax at 1 h, which was 35–50% among products with a designated Tmin of less than about 1 h, it is apparent that the true Tmin for these products is indeed less than 1 h. The Tmin for the low-protein meal was delayed, relative to the control, by about 1.5–2.0 h for brands E1 and E2, with similar delays for the Tmax. However, for brands E3, E4, and E5, the Tmin and Tmax were not significantly different from the control, indicating that the coatings of these products did not delay tablet disintegration in vivo, even though that was their intended purpose.

Three of the EC brands, E1, E2, E3, were also consumed with the high-protein meal. All three gave significantly lower bioavailability than the low-protein control and significantly lower bioavailability than the same product consumed with the low-protein meal. Brands E2 and E3 gave low bioavailability (<35%) with the high-protein meal. The Tmin and Tmax for E1 and E2 were increased (delayed) by 4–8 h relative to the control and by 3–5.6 h compared to consumption with the low-protein meal. Even for product E3, which gave no delay in the Tmin and Tmax for the low-protein meal, the high-protein meal caused a 1.5 h delay in the Tmin and a 2 h delay in the Tmax. For E1, the decreased bioavailability and greatly delayed (by 7 h) tablet disintegration time caused by the high-protein meal (54 g tuna in whole wheat sandwich plus milk, 31 g total protein), were not caused by the low-protein meal (no tuna, 5.5 g total protein). Prior studies with E1 showed that these effects were not caused by moderate protein meals (25 g tuna in whole wheat sandwich plus water, 13 g total protein; or 280 g yoghurt, 10 g total protein), where allicin bioavailability was 90–94%, the Tmax was 6–6.3 h and the delay in Tmax, relative to the control, was 2.9–3.1 h [17,37].

The alliinase activity of each product is also given in Table 6. For the six brands of enteric tablets consumed with the low-protein meal, there was no correlation between alliinase activity (26-fold range) and allicin bioavailability (2.9-fold range) (r^2^ = 0.07) or the Tmax (1.9-fold range) (r^2^ = 0.02). The products with both the highest (E1 and E4) and the lowest (E5) alliinase activity also gave the highest bioavailability, possibly indicating that the lowest observed alliinase activity was sufficient to allow for maximum conversion of alliin to allyl thiosulfinates. When E1, E2, and E3 were consumed with the high-protein meal, there was a moderate correlation (r^2^ = 0.92) between alliinase activity and allicin bioavailability, but it was not significant (*p* = 0.094). A correlation between type of enteric coating agent (Table 2) and allicin bioavailability could not be established. Brands E1 and E6 did not list their coating agents. Sodium alginate was used by the brands that gave both the highest (E5) and lowest (E3) bioavailability. Hence, the variation found among brands of enteric tablets appears to be more related to unknown manufacturing procedures than to coating agents or alliinase activity.

#### 3.7.3. Normal (Non-Enteric) Tablets

Allicin bioavailability from all of the normal tablets was found to be high, even though they disintegrated rapidly and their alliinase activity varied 30-fold (Table 6). Only the N1 tablets, when consumed with the high-protein meal, gave significantly lower bioavailability (73%) than the control; however, when an additional 225 mL of water was consumed with the meal, the bioavailability increased insignificantly to 85%, and the difference from the control disappeared. Even N4 tablets (same brand as N1), which were consumed 19 years after their manufacture, gave high bioavailability. Two brands, N1 and N2, were consumed with both the low-protein and high-protein meals. Unlike with enteric tablets, the high-protein meal did not significantly decrease allicin bioavailability. Normal tablets rapidly achieved maximum allicin production. The Tmin was not significantly different than the controls for any of the tests with normal tablets, while the Tmax was significantly longer only for N1 consumed with the high-protein meal and for the old N4, but the delay was only 0.6–1.3 h.

#### 3.7.4. Capsules

Although capsules were expected to give very low allicin bioavailability (<5%), due to their assumed rapid disintegration in stomach acid at a low pH, the three brands tested gave a broad range of bioavailability (26–109%, Table 6). For capsules containing fine particles (C1 and C2, Table 1), the difference in bioavailabilities (54% and 26%) appears to be due to differences in alliinase activity (10,840 and 210). However, products C2 and C3 had similar alliinase activity (210 and 250) with large differences in bioavailability (26% and 109%), even though they were made by the same manufacturer and had almost the same listed ingredients (Table 1 and Table 2). The only known difference between C2 and C3 is that C3 was substantially more coarse and darker in color (Table 1). It appears that the larger particle size of C3 offered more protection against gastric acid inhibition of alliinase, although such a dramatic protection seems unexpected. The results also indicate that alliinase activity as low as about 250 μg allicin min^−1^ g^−1^ garlic powder, if protected, is sufficient to allow for maximum possible allicin formation from alliin. Consumption of C1 and C2 with the high-protein meal, rather than with the low-protein meal, had no effect on allicin bioavailability. Capsules disintegrated and produced allicin almost as rapidly as the controls, with the Tmax values being an average of 0.35 h longer with the low-protein meal and 0.77 h longer with the high-protein meal.

### 3.8. Garlic Supplements: Gender Comparisons

No significant gender differences for allicin bioavailability or the Tmax were found for the raw homogenate control or for any of the supplements, with two exceptions: the Tmax when enteric E1 and E2 were consumed with a high-protein meal (Table 7). The Tmax is a close measure of their disintegration times, as breath AMS levels were undetectable for enteric tablets at 1–2 h before the Tmax was reached. Very long disintegration times (Tmax 11–24 h) were found with the high-protein meal for 4 of 6 women (av 18 h) and 1 of 7 men (11 h) consuming E1 and for 2 of 6 women (av 15 h) and 0 of 7 men consuming E2. When E1 and E2 were consumed with the low-protein meal, very long disintegration times were found only for 1 woman (18 h) consuming E1. Similarly, consumption of enteric-coated aspirin tablets with a meal (22 g protein, estimate) has been found to give substantially longer gastric residence times (8.4 h vs. 3.5 h) and plasma salicylate Tmin (10.8 h vs. 5.0 h) for women than for men (Tmax not reported). However, when the aspirin tablets were simply consumed with water and no food, both the gastric residence time (0.8 h), Tmin (2.7 h), and Tmax (8.3 h) were much shorter and showed no gender differences [52].

### 3.9. United States Pharmacopeia (USP) Dissolution Allicin Release (In Vitro) Compared to Actual Allicin Release (In Vivo)

#### 3.9.1. Enteric-Coated Tablets

The dissolution test (Table 8) revealed that the quality of the coating of all tested brands of enteric tablets was sufficient to prevent tablet disintegration after 2 h in acid, as required in the USP monograph [29]. In fact, all brands had the appearance of being undisturbed at the end of the 2-h acid stage, except that E4 tablets were swollen and blistered and E6 had one small blister per tablet. After entering the buffer stage (pH 6.8), three brands (E1, E2, E5) disintegrated rapidly, within the time specified (1 h) in the USP monograph, while the other brands (E3, E4, E6) disintegrated much slower, requiring 2.5–5.7 h. However, the average Tmax for breath AMS with the low-protein meal was similar for both sets of tablets (~4 h), indicating that they disintegrated at similar average rates in the body. Considerably higher dissolution allicin release (%) was found for brands with rapid disintegration times than for those with slow disintegration times (88 ± 5% vs. 5 ± 2% at 1 h in buffer or 88 ± 5% vs. 18 ± 9% at the time of complete disintegration), even though the average alliinase activity for both groups was similar. However, in vivo, there was a smaller non-significant difference between the same groups (88 ± 18% vs. 53 ± 18%, *p* = 0.07). Only one brand (E1) met the USP requirement of releasing at least 90% of the label claim (Table 1) for potential allicin.

For the rapidly disintegrating tablets (E1, E2, E5), there was moderately good agreement between percent dissolution allicin release and percent allicin bioavailability, when consumed with the low-protein meal (ratio near 1). Overall, the USP in vitro dissolution test was partly accurate for predicting in vivo results when enteric tablets were consumed with a low-protein meal, but not when consumed with a high-protein meal (Table 6). That is, when dissolution allicin release, as percent of allicin potential, was high (>80%), allicin bioavailability was medium to high (69–104%, av 88%) with the low-protein meal and moderate (32–57%, a 45%, Table 6) with the high-protein meal. Furthermore, for the slowly disintegrating tablets (E3, E4, E6), the actual allicin bioavailability (36–71%, av 53%) was about 11 times greater than that predicted (av 5%) by the USP dissolution method. Hence, the USP dissolution method is not reliable for predicting disintegration times in vivo or allicin bioavailability from enteric tablets.

#### 3.9.2. Normal (Non-Enteric) Tablets

Although the USP dissolution method was designed for enteric (delayed-release) tablets, it was prepared under the assumption that only acid-resistant enteric tablets would be able to provide high allicin release in the body. The method does present the general simulated gastrointestinal conditions to which all supplements are exposed. Hence, the same conditions were applied to normal tablets and capsules, where two hours of acid exposure should strongly inhibit alliinase activity and substantially decrease allicin release. As expected, all of the brands tested under dissolution conditions (N1, N2, N3) completely disintegrated in the 2 h acid stage (1.5–1.75 h) and dissolution allicin release was small (6–17%), even though alliinase activity ranged from low to high (Table 8). However, the in vivo allicin bioavailability averaged 95% with the low-protein meal, an average of eight times greater than expected based on the dissolution test.

#### 3.9.3. Capsules

Because capsules contain loose powder and shells that dissolve quickly, they were expected to undergo rapid disintegration, resulting in nearly complete inactivation of alliinase, and very low dissolution allicin release. The results for the three brands tested were partly unexpected (Table 8). Only C3 disintegrated rapidly in the acid stage (0.25 h), perhaps because it uniquely consisted of coarse particles, while C2 required the full 2 h in acid and C1 required the acid stage and 1 h of the buffer stage. Hence, C3 and C2 fully disintegrated in the acid stage, resulting in alliinase inhibition and undetectable levels of allicin release, as expected, while C1 released nearly half of its potential allicin.

Actually, the shells of C1 and C2 did disintegrate rapidly in the acid stage, but in both cases the capsule contents, rather than immediately disintegrating upon loss of the shell, congealed into a solid mass that disintegrated slowly. Three other brands of garlic powder capsules were also observed for their disintegration patterns in the acid stage (not shown); only one disintegrated rapidly (5 min; this product contained predominantly rice powder), while the other two also formed congealed masses that disintegrated slowly, by 30 and 60 min in acid. Hence, congealing of the garlic powder in the acid stage is a consistent pattern among garlic powder capsules comprising mainly fine garlic powder and is independent of other ingredients in the capsule; C1 listed no other ingredient (Table 2), yet it congealed the most. When tested in water, all four of the capsule products that had congealed in acid disintegrated about 4-fold faster; hence, the congealing is probably the effect of acid on the protein of fine garlic powder. In vivo, however, congealing appears to not occur. This conclusion is based on observation that similar times (5–12 min) were found for the occurrence of side effects associated with allicin release after capsule disintegration (garlic burp, heart burn, gastric disturbance) after consuming capsules C1 and C3.

There was not a consistent correlation between dissolution allicin release and allicin bioavailability of the capsules. Although the 42% allicin release for C1 agreed well with the 53% allicin bioavailability, the brands that gave undetectable allicin release gave 26–109% bioavailability. A large difference in disintegration times between C1 and C3 was found in vitro, but no difference was found in vivo, based on Tmax times for the high-protein meal.

### 3.10. Garlic Foods: Allicin Bioequivalence

Garlic foods prepared in the kitchen are usually made by the cooking of raw cloves or bulbs, such as by roasting or boiling or stir-frying, all methods of which completely inhibit alliinase. It is also common to simply dice (cut into small cubes) raw garlic and add it to various foods, a method which does not inhibit alliinase. Commercial garlic foods are normally treated with an organic acid or phosphoric acid, which acidity (pH 3.6–4.3) fully inactivates alliinase [14], especially under long-term storage. However, for garlic products that are cut into small pieces (the acid-minced and oil-chopped products), alliinase is active during chopping process, allowing for small to modest amounts of allyl thiosulfinates to form, depending upon the extent and coarseness of the chopping. After formation, the allyl thiosulfinates are gradually transformed at ambient temperature to allyl polysulfides.

An attempt was made to predict the amount of AMS produced by the body after consuming these garlic foods, especially when alliinase has been inhibited. It has been shown that allyl thiosulfinates account for >90% of the AMS produced when crushed raw garlic is consumed [17]. This was demonstrated by comparing the breath AMS produced upon consuming crushed raw garlic (all of the alliin previously converted to allyl thiosulfinates) with the AMS produced upon consuming pure allicin. Furthermore, it has been shown that allyl thiosulfinates and allyl polysulfides produce equimolar amounts of breath AMS [17]. Hence, it should be possible to predict the breath AMS-AUC for any garlic product, including alliinase-inhibited garlic, based on the content (Table 4) or expected yield (if alliinase is active) of allyl thiosulfinates and allyl polysulfides, relative to the raw garlic homogenate control. These predictions assume, as indicated from crushed raw garlic, that no other *S*-allyl compound present in garlic is metabolized to significant amounts of AMS. They also assume that the body does not contain significant endogenous alliinase activity. However, these predictions were found to be incorrect (Table 9). Typical concentration curves are found in Figure 4M–P.

As expected, nearly all of the alliinase-inhibited garlic foods produced substantially less AMS (smaller RF) than did the homogenized raw garlic control; the exceptions were oil-chopped and acid-minced garlic, due to their relatively high content of allyl polysulfides (Table 4). Nevertheless, the amount of AMS (AUC) produced from alliinase-inhibited garlic foods was 5–25 times higher than predicted (see ratio: actual/predicted)—except for oil-chopped garlic—giving an allicin bioequivalence of 14–34% for cooked garlic and 19–79% for acidified commercial products. Overall, these results demonstrate that the body can produce substantial amounts of AMS from *S*-allyl compounds in garlic by a mechanism that is independent of garlic’s alliinase activity, but this mechanism appears to be significant only when garlic’s alliinase activity is inhibited.

#### 3.10.1. Cooked Garlic

The intensity and duration of cooking made little difference in the allicin bioequivalence. Roasting at 215 °C (internal temperature 101 °C) for 60 min did not produce significantly less AMS than roasting at 160 °C (internal temperature 92 °C) for 30 min. Similarly, boiling (internal temperature 95 °C) for 45 min reduced allicin bioequivalence from 18% to only 14% (*p <* 0.05), compared to boiling for 4 min. However, the method of cooking made a difference. The roasted preparations gave about two times (*p <* 0.01) as much allicin bioequivalence as either of the boiled preparations. This was unexpected, as the internal temperature for roasting at 215 °C was higher than that which was found upon boiling. The difference is probably due to the higher content of allyl polysulfides in roasted garlic (Table 4).

#### 3.10.2. Acidified Garlic Products

The acid-minced and the oil-chopped commercial garlic products gave high allicin bioequivalence (66–79%) because of major tissue disruption before alliinase was inhibited by acidification. Both products were minced by an industrial process that was probably harsh (unlike the careful use of a razor blade for the diced raw garlic in Table 9). Based on visual examination, the oil-chopped garlic product also appears to have been partially crushed. These industrial procedures gave the products a desired and substantial garlic flavor. The comparatively low levels of alliin and high levels of allyl polysulfides in acid-minced and oil-chopped products (allyl polysulfides were 14% and 50% of the AADD, respectively, compared to <5% for pickled garlic, cooked garlic, and most of the supplements, Table 4) validate the substantial amount of tissue disruption that occurred in the preparation of these products. However, when undisturbed whole cloves were suspended in acid (pickled garlic), both allyl polysulfides and allicin bioequivalence were low. 

#### 3.10.3. Tmin and Tmax

Although the Tmin for the alliinase-inhibited garlic foods could not be determined (less than the first measure, 1 h), the % of Cmax achieved at 1 h provides an estimate of Tmin. To improve the accuracy of comparison to the control, the control values for % Cmax at 1 h and Tmax shown in Table 9 are for the “in sandwich” control (Table 5, test 3), rather than for the “in capsules” control (the standard for AUC and RF), because the garlic foods were also consumed inside sandwiches; the % Cmax at 1 h and Tmax are dependent on the initial time of body contact, which is delayed when consumed in capsules. The % Cmax at 1 h for the garlic foods was found to be 19–31%, indicating that AMS production began about 30 min after consumption. The Tmax for almost all of the garlic foods (3.1–4.4 h) was significantly longer than that of the in-sandwich control (1.3–1.8 h, Table 9). This difference was expected, due to (a) the lack of allicin and alliinase activity in the garlic foods, (b) the immediate contact of allicin with the body (starting in the mouth) for this control, and (c) the fact that allicin is lipophilic, readily passing through epithelial cell membranes, in contrast to the lipophobic alliin and γ-glutamyl-*S*-allylcysteine, the main *S*-allyl sources in the garlic foods. The similarity among the Tmax values for the alliinase-inhibited garlic foods indicates that they all have a similar and moderately effective mechanism by which AMS is produced.

#### 3.10.4. Diced Raw Garlic

For finely diced raw garlic, where active alliinase and about 97% of the initial alliin content (prior to cutting) were still present, predicting the amount of AMS production is not as straightforward as it is with crushed or homogenized raw garlic. This is because of the unpredictable amount of allyl thiosulfinates produced by alliinase upon chewing the diced garlic (about 60 cubes) in the bread and butter sandwich. It appears that consuming the sandwich caused only a small percent of the garlic cells to have ruptured, exposing alliin to alliinase. When the seven participants were asked if they felt a “garlic burn” in the throat, which burn is caused by allicin, only one person reported a noticeable burn, one that was mild. In a separate test, when a single cube was chewed without other food, there was an immediate and strong burn. Hence, only minor crushing of the garlic cubes occurred during consumption of the sandwich. 

The significantly longer Tmax for the diced garlic, compared to the control (3.1 vs. 1.3 h), and its high allicin bioavailability, demonstrate that garlic alliinase was active enough to convert most of the alliin to allyl thiosulfinates after entering the stomach. This was possible because the gastric pH was likely elevated to at least 4.0 for about 0.5 h by the buffering effect of the meal [28]; alliinase has some activity at pH 4.0 and maximum activity at pH 4.5 [14]. The amount of AMS produced upon consuming the diced garlic was close to that which was predictable based on the assumption that all of the alliin was converted to allyl thiosulfinates (ratio: actual AUC/predictable AUC = 0.8, Table 9). This confirms prior evidence that alliin-derived compounds account for nearly all of the AMS produced from garlic products in which active alliinase is present [17]. 

#### 3.10.5. Black Garlic

Black garlic is produced by incubating garlic bulbs at medium heat (60–90°C) and high humidity (80–90%) for 30–40 days, a process loosely referred to as “fermenting” or “aging,” during which the cloves turn black due to the Maillard reaction [53,54]. The process eliminates the sharp taste (mainly due to allicin) associated with raw garlic and replaces it with a sweet taste due to the reactions between sugars and free amino acids. Because of the virtual absence of AADD in black garlic (Table 4), the relative production of AMS from this garlic food gave an impossibly high value for RRF (285/7.2 = 3960%; RF = 228/0.8 = 285). If the allicin bioequivalence for black garlic is based on the remaining known *S*-allyl compounds in garlic (G/SAC), it would give a high RRF2 value (4.87/6.8 = 72%; RF2 = 228/46.8 = 4.87), which also seems excessive and indicates the presence of unknown *S*-allyl compounds. Because whole garlic bulbs were heated for a long time, alliinase was never significantly activated; hence, the absence of alliin in black garlic indicates that alliin was converted to new compounds unrelated to allicin, the identity of which is unknown. 

If the new compounds could be identified and quantified, the TKSA value would increase, giving lower values for RF2 and RRF2. It is possible to estimate the amount of new *S*-allyl compounds produced, based on the alliin loss. Although the initial content of alliin in the bulbs used to prepare the black garlic is not known, if one assumes it to be the average value among 46 garlic varieties, 55 μmol alliin/g fresh weight [49,50,51], then the amount of new *S*-allyl compounds generated from alliin would have been 561 μmol (55 × 10.2 g consumed). Hence, the new value for TKSA would be 608 μmol (47 μmol G/SAC plus 561 μmol new *S*-allyl compounds), giving a much lower RF2 (228/608 = 0.38) and much lower allicin bioequivalence of 6% (100% × 0.38/6.8). Furthermore, when allicin bioequivalence was estimated based simply on the weight (RRF3) of black garlic consumed, it was found to give a value of 5% (22.4/448), as calculated from the product RF3 (228/10.2 g = 22.4) and the control RF3 (394/0.88 g = 448). The RRF3 values for both roasted garlic products (35% and 32%, respectively) and both boiled garlic products (17% and 17%) were similar to their RRF2 values (Table 9), indicating the validity of estimating allicin bioequivalence on a weight basis. The agreement between RRF3 and the new estimated RRF2 indicates that the allicin bioequivalence of black garlic is close to 5% and that the new alliin-derived compounds are only poorly metabolized to AMS.

The new *S*-allyl compounds are most likely the products of the Maillard reaction [53] between reducing sugars and alliin. The Maillard reaction may have also occurred to a lesser extent with G/SAC. A 25% loss of G/SAC has been reported when black garlic is produced [53]. Further evidence for a decrease in the content of G/SAC during the manufacture of the black garlic used in the current study is that its content (4.5 μmol/g) is below the range found for 13 varieties of garlic (26.2 ± 9.0 μmol/g fresh wt, range 8–38) [49].

#### 3.10.6. Aged Garlic Extract 

Also tested was a commercial aged garlic extract supplement (not shown) that has some similarities to and differences from black garlic. It is sold under the name of Kyolic Aged Garlic Extract and is manufactured by Wakunaga of America, Mission Viejo, California, USA. Both black garlic and Kyolic AGE are promoted as “aged” garlic products; both contain *S*-allylcysteine (derived from GSAC) as their main identified *S*-allyl compound; neither contains active alliinase; and both contain little or no alliin or alliin-derived thiosulfinates or sulfides. While black garlic consists of whole bulbs incubated in heated, moist air for 30–40 days, Kyolic AGE is made by incubating sliced raw garlic in 20% ethanol for up to 20 months at ambient temperature [55,56]. The extract has been used in a large number of experimental and clinical studies.

A single test for allicin bioequivalence was conducted, in which nine tablets of Kyolic Formula 100 were consumed with the standard meal. The product label claims that each tablet contains 300 mg of “Aged Garlic Extract Powder (bulb)”. The composition of the product (same lot number) has been previously reported [37]. Nine tablets contained 0.86 μmol alliin, 16.8 μmol SAC, and 12.4 μmol GSAC, but no allyl sulfides (<0.2 μmol), giving a TKSA value of 30.1. The breath AMS curve revealed a Tmax of 2 h (Cmax = 8 ng/L; 31% of Cmax at 1 h), a plateau at Tmax that lasted about 10 h before returning to baseline at 20 h, and an AUC of 92 ng-h/L. The RF2 was 3.1 (92/30.1), while that for the control was 8.3 (543/65.5, Table 10), giving an allicin bioequivalence (RRF2) of 37% (3.1/8.3), which was higher than expected. When allicin bioequivalence was based on the weight of product consumed (RRF3), it was found to be only 2% (34/1551), as calculated from the product RF3 (92/2.7 g dry garlic = 34) and the control RF3 (543/0.35 g dry wt = 1551). As with black garlic, the large discrepancy between RRF2 and RRF3 (37% vs. 2%) indicates that much of the *S*-allyl (mainly alliin) lost during the aging process was converted to unidentified *S*-allyl compounds that were partly metabolized to AMS.

### 3.11. Special Garlic Extracts and Pure Compounds: Allicin Bioequivalence

The unexpectedly high results found for the allicin bioequivalence of alliinase-inhibited garlic foods (Table 9) indicates that the body itself contains either (a) substantial alliinase activity; (b) the ability to partially reactivate inactivated garlic alliinase; or (c) enzymes that can metabolize the *S*-allyl compounds in garlic (alliin, GSAC, SAC) to AMS without using allicin as an intermediate. In order to examine these possibilities, AMS production was examined after consuming pure alliin or pure SAC, as well as extracts in which the protein had been removed (PFHA) or the alliin selectively removed (AFHG). The results are summarized in Table 10.

#### 3.11.1. Alliin

After consuming l(+)-alliin in a sandwich, breath AMS was detectable at 0.2 h, reached Cmax at 3–3.5 h and gradually decreased almost to baseline by about 27 h (Figure 4S). About 5% of the alliin was metabolized to AMS (RRF2). This represents the maximum contribution that intestinal bacterial alliinase could make to in vivo production of allicin from alliin. Two tests with synthetic l(±)-alliin were also conducted with the same person who consumed l(+)-alliin (not shown). No significant differences in the Tmax, RF2, or RRF2 values were found between natural l(+)-alliin and synthetic l(±)-alliin.

#### 3.11.2. *S*-Allylcysteine (SAC)

After consuming SAC, breath AMS (measured initially every 30 min) was undetectable until 2.5 h, reached Cmax at about 8 h and gradually decreased almost to baseline by about 35 h (Figure 4T). About 6% of the SAC was metabolized to AMS (RRF2). The difference in Tmin between alliin (0.2 h) and SAC (2.5 h) indicates a difference in metabolic pathways, which is even more prominent in the difference in the Tmax values (3.2 h vs. 8.4 h; Table 10). In a previous report, breath AMS was not detectable after consuming a similar amount of AMS (730 μmol) because the method used was about 20 times less sensitive [17].

#### 3.11.3. Diallyl Sulfide (DAS)

Diallyl sulfide (DAS), a monosulfide, is not present in crushed raw garlic [21,37]. It is typically less than 3% of the small amount of sulfides found in garlic powder products (Table 4), and it is only about 2% of the large amount of sulfides found in commercial steam-distilled garlic oil [21]. Although its abundance is minor in garlic products, it has been included in Table 10 because it has been used in a large number of experimental studies [57,58]. After consuming DAS, breath AMS was undetectable until 4 h, reached Cmax at about 6 h and remained at Cmax (plateau) for about 23 h, and became undetectable at about 35 h (not shown). About 3% of the DAS was metabolized to AMS (RRF2). Possible impurities in DAS that are known to be fully metabolized to AMS are diallyl disulfide and allyl mercaptan [17]; however, analysis of the DAS used in the study revealed that allyl mercaptan was undetectable (<0.037%) and that the diallyl disulfide content was only 0.04%. Hence, impurities are not responsible for the breath AMS found after DAS consumption. A previous failure to detect AMS in the breath after DAS consumption was due to the use of a less-sensitive detector [17].

#### 3.11.4. Protein-Free, High-Alliin Garlic Extract (PFHA)

It was shown in Table 9 that AMS production from alliinase-inhibited garlic products is much greater than expected, based on the amount of alliin-derived dithioallyl compounds present. One possible explanation for these results, although unlikely, is that the body might be partially re-activating alliinase. To examine this possibility, an extract (PFHA) was prepared in which 97% of the protein of boiled garlic was removed, without reducing alliin. Alliinase is among the most abundant proteins in garlic and constitutes about 10% of the total clove protein [25,59]; hence, removal of 97% of the protein likely removed virtually all of the alliinase, and any amount remaining would have been inactive. Upon consuming PFHA, breath AMS was detectable in less than 0.25 h, reached 48% of Cmax in 1 h, reached Cmax in 3.6 h and remained at Cmax until about 8 h after consumption, followed by a gradual decline to near zero by 30–32 h (Figure 4Q). About 15% of the TKSA (RRF2) was metabolized to AMS (Table 10). Because alliinase and alliin-derived *S*-allyl compounds were absent in this product, and because GSAC and SAC have been shown to be metabolized to AMS at equal efficiencies (see product AFHS), the expected AUC, based on the response factors for pure l(+)-alliin and pure SAC and their content, would be 152 ng-h/L (399 × 0.38) for alliin plus 35 ng-h/L (71.4 × 0.49) for SAC, for a total of 187. In reality, the AUC was found to be 2.5-fold higher (472 ng-h/L). Similar enhanced ratios were found for boiled garlic, roasted garlic and pickled garlic (av 2.4, not shown). The nature of this enhancing factor is unknown, but it is unlikely to be a protein. The similarity found in the RRF2 values for PFHA (14.7) which contains almost no protein, and boiled garlic (14–18, Table 9), where protein has not been removed, demonstrates that the inactivated alliinase present in these garlic foods was not reactivated in the body to a measurable extent.

#### 3.11.5. Alliin-Free, High Γ-Glutamyl-*S*-Allylcysteine (GSAC) Extract (AFHG)

An extract (AFHG) void of alliin and alliin-derived compounds was prepared for the purpose of determining the effect of the remaining known *S*-allyl compounds (G/SAC) in garlic on breath AMS production. AFHG and PFHA were made from the same small batch of bulbs and extracted at the same ratio (14 mL water/g clove). Different amounts were consumed (57 mL and 68 mL) to obtain the same amount of G/SAC. Similar to consuming PFHA, consumption of AFHG resulted in breath AMS that was detectable in less than 0.25 h, reached 50% of Cmax in 1 h, and reached Cmax in 2.9 h, followed by a gradual decline to near zero at about 24–25 h (Figure 4R). The loss in AMS production, due to removal of alliin in preparing AFHG, was determined by comparing the allicin bioequivalence (RF3) of PFHA and AFHG on the basis of the weight of garlic clove in the consumed extracts: 4.9 g for PFHA and 4.1 g for AFHG. The RF3 for PFHA was 97 (AUC 472/4.9 g), while the RF3 for AFHG was 50 (AUC 203/4.1 g). Hence, only about 50% (50/97) of the allicin bioequivalence of PFHA was lost upon removal of alliin, demonstrating that about 50% of the AMS production from PFHA was due to G/SAC and that the body is capable of metabolizing significant amounts of G/SAC to AMS when alliinase is inhibited. 

Because GSAC and SAC are the only known *S*-allyl compounds in this extract, and because they have been shown to be metabolized to AMS at equal efficiencies (see product AFHS), the expected AUC for AFHG was calculated—based on the response factor for pure SAC and the content of G/SAC—to be 35 ng-h/L (70.6 × 0.49). In reality, the AUC was found to be 5.8-fold higher than expected (203 ng-h/L, Table 10), giving a response factor (RF2) of 2.85 for G/SAC when they are present in garlic, compared to an RF value of 7.2 for the allyl thiosulfinates in the raw homogenate control (Table 9). As with PFHA and alliin, something is enhancing the metabolism of G/SAC to AMS in the absence of active alliinase, and it is more effective in doing so for G/SAC than it is for alliin.

#### 3.11.6. Alliin-Free, High-SAC Extract (AFHS)

The kinetic difference between AMS production from GSAC or SAC in garlic was examined after converting 95% of the GSAC in AFHG to SAC with added γ-glutamyl transpeptidase. The kinetics of the AMS concentration curve for AFHS was essentially identical to that of AFHG (Tmin <0.25 h, 43% of Cmax reached in 1 h, Cmax reached in 2.5 h, followed by gradual decline to near zero in 23–24 h). Both products gave the same RRF2 value (Table 10). Hence, GSAC and SAC are metabolized to AMS with equal efficiency, which also indicates that the body is highly efficient at cleaving GSAC to form SAC. Surprisingly, conversion of the GSAC to SAC did not change the Tmax (2.4 h), even though the Tmax for pure SAC was 8.4 h. Hence, the factor that is enhancing the amount of SAC being metabolized to AMS is also enhancing the rate of AMS formation from SAC.

#### 3.11.7. AFHG Plus Added SAC

To test the proposal that something in enzyme-inactivated garlic is enhancing the percent of metabolism of SAC to AMS, pure SAC was added to AFHG, followed by consumption. Because the addition of 141 μmol pure SAC to the AFHG increased the total SAC content three-fold (from 70.6 to 212 μmol), the AUC for this product was expected to be three-fold higher than for AFHG alone, or 636 ng-h/L (3 × 212). In reality, the AUC was found to be only 277 ng-h/L (Table 10), which is only about 30% higher than for AFHG. This low increase is either due to a highly non-linear dose response or something else is involved. 

Although AFHG was consumed at only one dose, consumption of another alliinase-inhibited garlic product, acid-minced garlic (Table 9), at 7 g and 20 g, gave a fairly linear response (AUC/wt ratio: 1.2) in the AUC range of 740 to 2600 ng-h/L (not shown). Hence, non-linearity is unlikely to be the reason for the low response to the added SAC. The AUC value of 277 ng-h/L for AFHG + SAC is very close to the calculated value of 281 ng-h/L that is found if the added SAC was metabolized to AMS in an unenhanced manner [AUC from AFHG (212) and from added pure SAC (141 μmol × 0.49, RF) = 281)]. Hence, the factor in garlic that enhances the metabolism of SAC to AMS appears to be present only in sufficient quantity to enhance the amount of G/SAC naturally present in garlic, an unlikely situation.

#### 3.11.8. Search for Unknown *S*-Allyl Compounds

Rather than the proposed presence of an enhancing factor to explain the higher than expected amounts of breath AMS from alliin-free or alliinase-inhibited garlic, another explanation could be the possible presence of substantial amounts of previously undiscovered *S*-allyl compounds, such as peptides containing *S*-allylcysteine, *S*-allylcysteine sulfoxide (alliin), γ-glutamyl *S*-allylcysteine, or γ-glutamyl *S*-allylcysteine sulfoxide. To explore this possibility, both AFHG extract and roasted garlic (prepared at 160 °C) were incubated with a large excess of either protease or alkaline protease, followed by incubation with γ-glutamyl transpeptidase (see Section 2.21). Protease alone caused no change in alliin, SAC, or GSAC. Alkaline protease alone or γ-glutamyl transpeptidase alone caused complete depletion of the free GSAC present before incubation and increased SAC only by the amount expected from hydrolysis of the free GSAC, with no change in alliin. Combining the proteases with γ-glutamyl transpeptidase also did not increase the amount of SAC beyond that expected from hydrolysis of the free GSAC, also with no change in alliin. Hence, no hidden or unknown *S*-allyl sources were revealed by this hydrolysis protocol. Thus, the real reason for the enhanced metabolism of SAC to AMS in PFHA, AFHG, AFHS, and alliinase-inhibited garlic foods cannot be explained based on the present work and remains unknown.

### 3.12. Accounting for the AMS Formed upon Consumption of Alliinase-Inhibited Garlic Products

To determine the individual contribution of alliin and G/SAC to the breath AMS content after consuming alliinase-inhibited garlic foods, their in-garlic response factors were determined, in contrast to using the response factors found after consuming the pure compounds (0.38 for alliin and 0.49 for SAC, Table 10). Based on the results with AFHG, where GSAC and SAC are the exclusive known *S*-allyl compounds, the in-garlic response factor for G/SAC, in the absence of active alliinase, was found to be 2.85 (Table 10), a 5.8-fold enhancement (2.85/0.49). Based on the results with PFHA, where alliin is 85% of the known *S*-allyl compounds, the in-garlic response factor for alliin, after correcting for the amount of GSAC and SAC present, was calculated to be 0.67 (Table 11, footnote 2) when alliinase is inactive, a 1.8-fold enhancement (0.67/0.38), in contrast to a response factor of 7.2 when alliinase is active.

An important concern is whether or not enough is known about the composition and response factors for the *S*-allyl compounds in alliinase-inhibited garlic foods to be able to approximately predict the AMS-AUC values. As shown in Table 11, the average predicted AUC was 93% of the actual, compared to 19%, when only the alliin-derived allyl thiosulfinates and allyl polysulfides (AllylTS/Sx) were included. A range of 72 to 133% indicates that some of the actual AUC may be unaccounted for, especially for roasted garlic and acid-minced garlic, or that it is in the range of experimental or interpretive error. Alternatively, perhaps the in-garlic response factors for G/SAC and alliin are not the same in each of these garlic foods as it is for AFHG or PFHA, due to different methods of preparation. However, a prediction that is at least 72% accurate represents a reasonably good understanding of the *S*-allyl sources and their response factors.

Using the in-garlic response factors, it was found that alliin, AllylTS/Sx, and G/SAC accounted for an average of 36%, 19%, and 39% (total 94%) of the actual AUC. If all contribution from G/SAC had been eliminated, alliin and AllylTS/Sx would have accounted for only 55% of the actual AUC, demonstrating that GSAC and SAC are important contributors to breath AMS for alliinase-inhibited garlic products. Additionally, if the response factors found for pure SAC and pure alliin had been applied, G/SAC and alliin would have accounted for only 27%, rather than 75% of the actual AUC. This would have left most of the actual AUC unaccounted for and demonstrates the importance and approximate accuracy of the in-garlic response factors.

### 3.13. Accounting for the AMS Formed upon Consumption of Alliinase-Active Garlic Supplements

Because G/SAC contributed about 40% of the breath AMS found after consuming alliinase-inhibited garlic foods (Table 11), it can no longer be assumed that G/SAC makes no contribution to the AMS found after consuming garlic products that have active alliinase, even though SAC is known to not be a substrate for alliinase [26]. The data in Table 12, arranged in order of decreasing allicin bioavailability (RRF), attempts to explain the extent to which the various *S*-allyl sources contribute to the breath AMS found after consuming garlic supplements containing active alliinase. Determining the contribution of each *S*-allyl source is more complex than it is for alliinase-inhibited garlic foods. The complexity is mainly due to the different levels of protection of alliinase from gastric acid among supplements. Alliinase protection appears to vary with coating type, buffering capacity of the garlic powder, the influence of added excipients (Table 2) [30], particle size of garlic powders used in capsules (Section 3.7.4), and reversibility of alliinase activity inhibition when the local gastric pH does not go below 3.0–3.5 [14]. With enteric-coated tablets, additional variability is caused by the effect of meal protein content on allicin bioavailability (Table 6). A further significant complication is the unknown extent to which the metabolism of G/SAC to AMS is enhanced when alliinase activity is inhibited to variable degrees, which enhancement has been demonstrated to be 5.8-fold when alliinase is fully inactivated prior to consumption, as with cooked or acid-treated garlic foods (Section 3.11.5).

The contribution of each *S*-allyl source to the observed AMS-AUC has been determined based on the in-garlic response factors for alliin, allyl polysulfides, and G/SAC, but the values for these response factors for alliin and G/SAC are not straightforward. The RF for alliin has been calculated (Table 12, footnote 2) based on the RF for allyl polysulfides (7.2), which is independent of alliinase activity, and the RF for G/SAC, which value is uncertain, especially when alliinase inhibition is substantial. Two values are proposed in Table 12 for RF_G/SAC_: the unenhanced value of 0.49, which is used when the allicin bioavailability is >50% (above the line in Table 12), and a four-fold enhanced value of 2.0 when alliinase inhibition is substantial, but not complete, as indicated by allicin bioavailability <40%. The proposed RF_G/SAC_ value of 2.0 is less than the value found (2.85) when alliinase is completely inhibited. The in-garlic response factor for alliin (RF_alliin_) was found to vary for each product, but it generally declined according to the in vivo inhibition of alliinase, as indicated by the percent allicin bioavailability. The RF_alliin_ can also be used to calculate (Table 12, footnote 3) the percent conversion of alliin to AMS, which value closely reflects the percent allicin bioavailability.

When the allicin bioavailability was >50% (presumed RF_G/SAC_ = 0.49, *n* = 10), alliin and the small amounts of allyl polysulfides—formed during manufacture of the garlic powders—accounted for an average of 95% of the AMS-AUC. In these cases, the contribution from G/SAC was small (1–10%, average 5%), which was true for all brands of normal tablets, for 4 of 5 brands of enteric tablets consumed with the low-protein meal, and for 2 of 3 brands of capsules. When the allicin bioavailability was <40% (presumed RF_G/SAC_ = 2.0, *n* = 4), the contribution from G/SAC was elevated to 13–21% (av 15%), excluding capsule brand C2, which was exceptionally high at 77%, presumably due to its very low initial alliinase activity (Table 6), the fineness of the garlic powder, and perhaps to its exceptionally high content of G/SAC (Table 4). Hence, G/SAC is not a significant contributor to breath AMS from garlic supplements, except when inhibition of alliinase activity substantially reduces allicin bioavailability.

## 4. Discussion

### 4.1. Unexpected Allicin Bioavailability from Garlic Supplements—Meal Effects

Normal tablets and capsules were expected to disintegrate rapidly in the stomach under low pH conditions (pH < 3) in which alliinase would be fully inhibited, resulting in little (<5%) allicin bioavailability. On the contrary, all brands of normal tablets gave high allicin bioavailability even though they disintegrated rapidly and achieved Tmin and Tmax as rapidly as the raw garlic control capsules containing pre-formed, acid-stable allicin (Table 6). Supplement capsules also provided much higher allicin bioavailability than expected and did so almost as rapidly as the control, but the range in bioavailability was broader for the capsules (26–109%) than it was for the normal tablets (73–111%). Unlike the enteric tablets, allicin bioavailability for normal tablets and capsules was the same with both the low- and high-protein meals. Certainly, the main reason for these high results is the temporary buffering effect of the meals upon the gastric pH. It has been shown that when a person begins to consume a meal that the basal gastric pH (1.4–1.9) rapidly increases in about 14 min to a maximum of pH 4.4–6.7 (median 6.3) and that it requires an additional average of 106 min before gastric acid secretion can overcome the buffering effect of the meal and return the gastric pH to the basal level [27,28]. Garlic alliinase is inactive at pH 3.5 or below, has some activity at pH 4.0, and has maximum activity at pH 4.5–6.5 [14,60]; hence, when the gastric pH is at 4.0 or above alliinase will be active. Studies with typical meals for breakfast, lunch, and dinner have shown that the gastric pH is ≥4.0 for an average of 0.7 h (0.3–1.2 h, *n* = 6) after consumption [27,28,42]. Even consumption of a cup of tea or coffee can almost raise the gastric pH to 4.0 for about 5 min [28]. Complete formation of allicin from alliin in garlic powder suspended in aqueous medium takes place very rapidly: in 0.2 min at pH 6.3 and in 10 min at pH 4.5 [14]. Hence, both low-protein and high-protein meals would provide a gastric pH ≥ 4.0 for an ample amount of time for the alliinase in disintegrated normal tablets and capsules to convert most of the alliin to allicin in the stomach.

For enteric-coated tablets, the type of meal with which the tablets were consumed had a substantial effect on allicin bioavailability and tablet disintegration time (Table 6). This was true for all three of the brands that were consumed with both meal types. Compared to consumption with the low-protein meal, the high-protein meal caused an additional delay in the Tmin by 2–6.5 h, an additional delay in the Tmax by 2.3–5.6 h, and a decrease in allicin bioavailability from an average of 65% to an average of 37%. Similarly, a high-protein meal (compared to no meal) has been shown to substantially delay the disintegration of enteric-coated aspirin tablets by delaying the gastric emptying time [52,61,62]. When enteric aspirin was consumed with a high meat meal (58–70 g ham, 33–36 g protein, estimate), rather than with water only, the Tmin for plasma salicylate increased from 2 h to 5 h and the Tmax increased from 6 h to 9 h, indicating a 3 h delay in gastric emptying time [61]. When enteric aspirin was consumed with a meal of fried egg, milk and cheese (22 g protein, estimate), rather than with water only, the gastric residence time was found to increase from 0.8 h (range 0.25 to 1.5 h) to 5.9 h (range 3 to 12 h), with increases in Tmin of 5.2 h and Tmax of 5.5 h [52]. The reason these high-protein meals delayed the gastric emptying time for enteric aspirin is related to the fact that food must be digested to particle sizes of 1 mm or less in order to exit the stomach through the pyloric sphincter, but it takes several hours for this amount of digestion to occur for meat chunks and other foods that are slow to digest [42]. Once the food has been sufficiently digested and expelled from the stomach, non-digestible objects, such as enteric tablets, are expelled by the final sweep of the interdigestive myoelectric contraction [42,63].

Enteric tablets are designed to resist a low gastric pH for at least 2 h, but when consumed with a high-protein meal, the tablets will be exposed to a low pH for about 6 h and perhaps as long as 12 h, which probably makes the coating vulnerable to weakening, swelling, and possibly to partial disintegration, allowing gastric fluid of a low pH to come in contact with and at least partially inhibit alliinase. A second reason why a high-protein meal contributes to decreased allicin bioavailability is due to a temporary rise in the gastric pH, caused by the buffering effect of meal protein, as mentioned previously, to a range (4.4–6.7) in which enteric coatings are meant to dissolve, 5.0–7.0 [64]. Hence, some compromise in the integrity of the coating would be expected to occur before the pH of the gastric juice returns to a low value and remains low for several hours, during which time it can penetrate the damaged tablets, inhibit alliinase, and reduce subsequent allicin formation in the small intestine.

The low-protein meal used in this study contained no meat, egg or cheese; therefore, it would have been digested much more rapidly than the high-protein meal, and the tablets would have passed rapidly through the stomach, perhaps at a rate similar to the 0.8 h gastric residence time observed when enteric aspirin tablets are consumed with water only (fasting) [52]. The rapid passage of E1 and E2 through the stomach when consumed with the low-protein meal is indicated by a Tmin for breath AMS of 2.2–2.6 h, which is similar to the Tmin of 2–2.7 h found for plasma salicylate when enteric aspirin tablets were consumed with water only [52,61]. The short gastric residence time would expose the tablets to a low pH for less time, with less likelihood for decay of the enteric coating and less inhibition of tablet alliinase activity. Furthermore, the smaller amount of protein in the low-protein meal would also mean less buffering of the basal gastric pH, a lower temporary maximum pH in the stomach (perhaps no higher than 4–4.5), less compromising of the integrity of the coating, less inhibition of garlic alliinase activity in the stomach, and higher production of allicin in the small intestine, as was observed with most of the enteric products tested in this study.

The only known food component that interacts with allicin at body temperature is protein-derived cysteine. Allicin reacts quantitatively with cysteine, to form two moles of *S*-allylmercaptocysteine (allyl-S-S-Cys) [16,32]. This reaction probably occurs to some extent when the allicin released from garlic products in the gastrointestinal tract comes in contact with the cysteine released from digested meal protein. The formation of *S*-allylmercaptocysteine would increase with increased meal protein content. However, this reaction probably has little effect on allicin bioequivalence, because *S*-allylmercaptocysteine has been shown in human studies to also be quantitatively metabolized to AMS, at the same rapid rate as for allicin [17].

### 4.2. Comparing Garlic Supplements and Garlic Foods through Allicin Metabolite Equivalence

Clinical trials that explore the possible health benefits of garlic typically use garlic powder supplements in order to provide a consistent, stable and convenient dosage form that is usually also standardized to a specific allicin potential and/or alliin content. A common and important concern among consumers is the amount of a preferred garlic food, such as cooked garlic, that one needs to consume to obtain the health benefits indicated for the supplements used in clinical trials. A similar concern is the amount of a preferred supplement one needs to consume to obtain the possible health benefits reported for a different supplement. These concerns can be addressed by comparing their allicin metabolite equivalence through the AMS-AUC. This comparison assumes that allicin or its metabolite(s) are responsible for most of the observed health benefits of a garlic product. 

Table 13 gives dose comparisons among garlic supplements and garlic foods based on allicin metabolite equivalence, compared to consuming 2 g of crushed raw garlic or three N1 tablets. Two grams of raw garlic is half of the therapeutic dose (4 g) recommended by the German Commission E [65]. Also, most clinical trials with garlic powder supplements have used the equivalent of 1–2 raw garlic, based on claimed alliin content of 7.8–17 mg or equivalent allicin potential of 3.6–7.8 mg [1]. The allicin potential of raw cloves varies from about 2.4 to 4.6 mg/g fr wt, with an average of 3.6 ± 0.9 mg/g fr wt [19,20,30,66]. The raw garlic control used in this study and shown in Table 13 had an allicin potential of 3.97 mg/g clove or 7.9 mg for 2 g. Hence, the raw garlic control contained a typical amount of allicin. Product N1 has also been shown in Table 13 for comparisons because it is the main product that has been used in clinical trials with garlic supplements, even though the results with N1 have been inconsistent [1,2,3,67]. Three tablets has been the most common dose used in these trials. The N1 tablets in these trials have been consistently standardized to contain 3.9 mg alliin/tablet and to yield 1.8 mg allicin/tablet [1,68,69,70]. Three N1 tablets represent about 1 g of typical raw garlic, with respect to alliin content (11.7 mg per 3 tablets vs. the average for raw garlic, 9.7 ± 2.0 mg/g fr wt [49,50,71]) and with respect to allicin metabolite equivalence (400 vs. 435 ng-h/L, Table 13). 

Among garlic supplements (Table 13), there is a large range in the amount required to obtain the same allicin metabolite equivalence as 2 g raw garlic (0.7–27 tablets/capsules) or three N1 tablets (0.3–12 tablets/capsules). This range is due to variation in tablet/capsule size (0.1–1.0 g garlic powder, Table 1), variation in alliin content (6.5–49 mg/g garlic powder, Table 4), and variation in allicin bioavailability (RRF, 22–111%, Table 6); it highlights the need to carefully evaluate garlic supplements being considered for use in clinical trials. For alliinase-inhibited garlic foods, 5.9 g of roasted garlic and 11 g boiled garlic must be consumed to obtain the same equivalence as 2 g raw garlic. These are not unreasonable amounts, because it is often less objectionable (no throat burn, caused by allicin release from raw garlic) for most people to consume 6–11 g (2–3 average cloves) of cooked garlic than to directly consume 2 g of raw garlic. At 2.6–5.2 g, cooked garlic provides the same equivalence as three N1 tablets. For the three acidified commercial garlic foods (pickled, acid-minced, oil-chopped), 5.3 to 19 g or 2.4 to 8.6 g provide the same equivalence as 2 g raw garlic or three N1 tablets.

### 4.3. Garlic Supplements Used in Former Clinical Trials: Evaluation of Their Allicin Bioavailability

When clinical trials with garlic products show positive effects with some products and null or fluctuating effects with other products, it is reasonable to question both the dose and the availability of the suspected active compound(s), especially because of the tenuous nature of allicin formation. Hence, the main garlic supplement (Kwai, N1) used in clinical trials on the possible cardiovascular effects of garlic has been criticized as not representing raw garlic, due to low allicin release found in vitro under USP-defined simulated gastrointestinal conditions [31]. However, upon evaluating allicin bioavailability from this garlic supplement in vivo through breath AMS production (Table 6), it was found to give high allicin bioavailability that was not significantly less than that of crushed raw garlic, regardless of meal type. Even Kwai tablets that were manufactured in 1992, in the middle of the time period that most of the clinical trials with these tablets were being conducted (1986–2000), were found to give equally high allicin bioavailability 19 years later (Table 6, product N4). Hence, the results of the many clinical trials that have been conducted with this consistently standardized garlic supplement can be considered as valid for representing crushed raw garlic. However, the frequently used daily dose of 3 Kwai tablets represents only a modest amount of typical raw garlic, 0.92 g (Table 13).

Garlicin (product E1), an allicin-standardized enteric tablet, has been used in a 2007 clinical trial that found no effect on serum lipids at a daily dose of 4 tablets, representing 4 g of raw garlic [72]. The product has been previously reported [37], and is again reported (Table 6) to have high allicin bioavailability, equivalent to that of crushed raw garlic. Hence, the results of this 2007 trial do represent a high dose of raw garlic. The only other garlic supplement that this study has investigated for allicin bioavailability that has also been used in a clinical trial is product N3 (GNC 1000), which consists of non-standardized non-enteric tablets. A clinical trial using this supplement at 2 tablets per day concluded that “garlic” has no effect on platelet function in vivo [73]. Even though it was presumptuous, not knowing the quality of the supplement, to extrapolate the results of the trial to garlic itself, the allicin bioavailability of the product was found to be high and equivalent to that of crushed raw garlic (Table 6). Additionally, the product had a high alliin content, such that 2 tablets represented a dose of 5.7 g of typical raw garlic (Table 13). Therefore, it can be confidently concluded that this clinical trial found no effect on platelet function by a garlic supplement that accurately represented a high dose of raw garlic.

### 4.4. Possible Mechanisms for AMS Production from Alliin and GSAC and SAC (G/SAC) in the Absence of Active Garlic Alliinase

**Figure 6:**
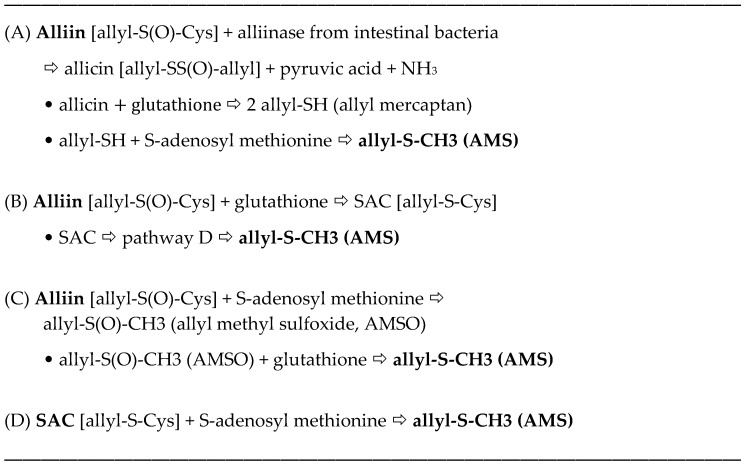
Possible metabolic pathways for the partial transformation of alliin and SAC (*S*-allylcysteine) to AMS (allyl methyl sulfide) upon consuming alliinase-inhibited garlic. For structures, see Figure 1 and Figure 2. Of the three proposals for alliin, only pathway C is likely to be relevant.

One of the remarkable outcomes of this study has been the finding of unpredictably high allicin bioequivalence for alliinase-inhibited garlic foods (Table 9), as well as for garlic extracts from which alliin and alliin-derived compounds were selectively removed (AFHG, AFHS, Table 10), leaving G/SAC as the only source for AMS production. Hence, the body has metabolic pathways that lead to partial transformation of alliin and G/SAC to AMS without garlic alliinase. Three possible pathways for alliin and one pathway for G/SAC are presented in Figure 6.

Alliin pathway A. Since shortly after the discovery of alliin, it has been known that some bacteria, including some that are commonly found in the intestinal tract, possess alliinase activity [26,74,75,76]. It is also possible body organs could possess alliinase activity, although none has yet been reported; however, human serum has been shown to contain antibodies to alliinase [77]. As reported in Table 10, it was found that consumption of pure alliin resulted in only 4.6% conversion (allicin bioequivalence) to AMS. This small value represents the total contribution to AMS production from alliin through (a) intestinal bacterial alliinase; (b) any alliinase activity that the body organs might possess; and (c) non-alliinase metabolism of pure alliin to AMS. Hence, bacterial alliinase activity transforms only 0–4% of the alliin to allicin.

Alliin pathway B. One proposed pathway for the body to metabolize alliin to AMS without allicin as an intermediate is glutathione reduction of alliin to SAC, followed by metabolism of SAC to AMS by pathway D. However, this pathway would result in alliin being metabolized to AMS slower than SAC to AMS, but the opposite was found to be true, with the Tmax for pure alliin being 3.2 h and the Tmax for pure SAC being 8.4 h (Table 10). Furthermore, a study with dogs fed pure alliin has shown rapid and essentially complete absorption, but less than 0.1% of the administered alliin was found in the blood or urine as SAC or N-acetyl-SAC, while the 22% of the dose was found in the urine as *N*-acetyl-alliin, 6% as alliin, and 72% as unidentified compounds [78]. It is assumed that dogs and humans metabolize alliin similarly because they have been shown to metabolize SAC similarly [79]. Hence, the body is probably not capable of reducing alliin to SAC. Therefore, pathway B probably does not occur to a significant extent in humans.

Alliin pathway C. Another proposed pathway from alliin to AMS, one without allicin or SAC as an intermediate, involves methyl transfer from *S*-adenosyl methionine (SAM) to the allyl-S(=O) group of alliin to form allyl methyl sulfoxide (AMSO), followed by glutathione reduction of AMSO to AMS. Formation of AMSO as a metabolite of an *S*-allyl compound derived from garlic was first demonstrated in rats fed diallyl disulfide, in which AMS and greater amounts of AMSO and AMSO_2_ (allyl methyl sulfone) were found in the liver, plasma, and urine [80]. Both rat and human liver microsomes were shown to oxidize AMS to AMSO and AMSO_2_ [81]. Allicin follows the same metabolic pathway as diallyl disulfide, as both are found to produce the same amount of human breath AMS when consumed as pure compounds [17]. AMS, AMSO, and AMSO2 have recently been found in the serum of rats fed aqueous garlic homogenate [82]. Hence, allicin is expected to be metabolized to AMS, AMSO, and AMSO_2_.

It also appears that alliin, in the absence of active garlic alliinase, is partially metabolized to AMS, AMSO, and AMSO_2_. Recent studies have shown that when people consumed raw garlic cubes (3 mm cubes) that AMS, AMSO, and AMSO_2_ were rapidly formed and found in breast milk (Tmax about 2.5 h) and urine (Tmax about 1.5 h) [83,84]. Based on how these studies were conducted, however, there was little opportunity for alliin to be transformed by alliinase to significant amounts of allicin, even though there were three opportunities to do so. Preparation of the garlic cubes probably resulted in minimal damage to the cell walls, allowing for little contact between alliin and alliinase. The current study found that only 3% of the alliin content of raw garlic was transformed to allyl thiosulfinates when the garlic was cut (diced) into 3 mm cubes (Section 3.5 and Table 4). Chewing the cubes would have caused the breakdown of cell walls and rapid formation of allicin before reaching the stomach, but the cubes were swallowed without chewing (Personal Communication) [85]. Furthermore, the cubes were consumed without a meal (with water only) and, therefore, without the pH buffering effect of a meal. Hence, the gastric pH was probably near 2.0 when the garlic cubes entered the empty stomach [27,28], which is well below the pH at which alliinase is fully inactivated (≤3.5) [14]. Thus, garlic alliinase was probably inactivated in the stomach before alliin was released from the garlic cubes. This indicates that probably only a small percent of the alliin was ever transformed to allicin. Hence, the results of the garlic cube studies [83,84] mainly represent the metabolism of alliin in the absence of active garlic alliinase and validate the partial metabolism of alliin to AMSO in the body. 

SAC pathway D. The metabolism of pure SAC has been studied in dogs and humans, although no metabolites were initially found [79,86]. A more extensive study with dogs found SAC to be rapidly and completely absorbed, but only 13% of the administered dose of SAC was accounted for among the urinary metabolites: 12% as alliin plus *N*-acetyl-alliin and 1% as SAC plus *N*-acetyl-SAC [78]. Based on the presence of small amounts of breath AMS after consuming pure SAC (Table 10) and the mentioned finding of AMS, AMSO, and AMSO_2_ in the urine after human consumption of garlic cubes [84], it is most likely that a significant portion of the 87% unknown SAC metabolites includes AMS, AMSO, and AMSO_2_. The simplest pathway from SAC to AMS would be methyl transfer from SAM to the allyl-S group of SAC. Although evidence for this transfer is currently lacking, the fact that SAM is involved in a large number of different types of methyl transfers [87] makes this pathway a reasonable possibility. Because the methylation reaction involves SAM-dependent methylase, it might be possible to explain the large difference in response factors between pure SAC and the G/SAC in garlic (0.49 vs. 2.85, Table 10) based on enhancement of SAM-dependent methylase activity by a currently unidentified compound in garlic.

### 4.5. Effects and Side Effects of Odorous AMS

As a metabolite of allicin, AMS has been used in this study as a tool to determine the ABB from garlic supplements and garlic foods. However, AMS has also been demonstrated to have several biological activities, including reduction of induced lipid peroxidation in mice [88], antibacterial activity [89], inhibition of cytochrome P450 2E1 [90], reduction of induced colon cancer in rats through modulation of cytochrome P450 enzymes [91,92], and increased human breath acetone levels [17]. Addionally, both AMS and AMSOhave been found to decrease induced hypertrophy in rat hearts [82]. AMS is responsible for most of the well-known breath odor that persists for several hours after consuming raw garlic, which odor is often more noticeable by close associates than by the consumer [36,93]. AMSO and AMSO_2_ have no odor [83], nor have they been found in the breath, using methods that could have detected them [34,35,36], indicating that they are too polar to be transferred to the breath. Even though many garlic supplements have been found to produce as much AMS as raw garlic, based on allicin bioavailability (Table 6), sustained breath odor perception from supplements appears to be less than that from raw garlic. In a study in which 49 participants ate sandwiches containing 4 g of crushed raw garlic daily for six-months, 57% of the participants reported “bad body and breath odor … often or almost always”, while none of the 47 participants who consumed four enteric E1 tablets daily for six months reported an odor problem [72]; it is possible that many of the E1 participants noticed a garlic breath odor, but did not consider it to be strong enough to qualify as a bad odor. Due to the numerous clinical trials with N1 tablets, there have been many reports in relation to breath odor. In a study with 123 participants who consumed three tablets, each containing 300 mg of garlic powder (same dose as in Table 6), for 14 days, 20% reported moderate to strong breath odor, 25% reported slight odor and 55% reported no odor [94]. In another study with the same dose of N1 tablets for 16 weeks with 1997 participants, 4% reported strong breath odor, 14% a definite odor, and 38% a slight odor [95]. Other studies with N1 tablets at this same dose have reported garlic breath odor in 11–60% of the subjects [96,97,98,99]. In a clinical trial with N3 tablets (2/d), it was stated that no side effects were found, but it is likely that breath odor was not considered to be a side effect [73].

Of the 13 garlic supplements examined in this study, the package labels for almost all of them made some type of claim in relation to dramatic reduction of the breath odor normally associated with consuming raw garlic (Table 1). The claims include “odor free” (E1, E2, E4, E5, N2, N4) or “odorless” (E6, N3) or “no garlic breath” (E3) or “deodorized garlic” (C2). One product (C3) claimed that it was “odor controlled”. Terms such as “odor controlled” or “reduced odor” represent more accurate statements that should be applied to most of the supplements, including some of the enteric products where the time before the appearance of AMS in the breath has been delayed, compared to consuming raw garlic (Table 6). Only two products made no claims related to odor: N1 and C1. The package insert to product N1 states that a side effect can be odor from the breath or skin, but it also states that the odor is linked to the product’s effectiveness and recommends that the daily dose of three tablets be consumed as one tablet with each of the three main daily meals in order to reduce the eventual breath odor. Division of the recommended daily dose over 2–3 meals is also recommended on the labels for most of the products in Table 1, except for four brands of enteric tablets where the daily dose is only one tablet. Because the time to reach maximum breath AMS concentration (Tmax) is about 2–4 h for many of the supplements (Table 6) and because the breath AMS concentration rapidly declines after the Tmax (Figure 4), dividing the recommended dose over 2–3 meals compared to consuming all at one meal will certainly decrease the concentration of AMS in the breath and decrease the perceived breath odor.

### 4.6. Recommendations

Now that the bioavailability of allicin in humans has been determined through its metabolite, AMS, for a variety of types and brands of garlic supplements of known composition under two meal conditions, more accurate recommendations can be made for clinical trials and garlic supplement manufacturers than has been made or assumed in the past. These recommendations apply to garlic supplements that are intended to represent raw garlic in dried form. They assume that the possible health benefits being sought are mostly related to alliin-derived allicin.

#### 4.6.1. Alliin Content or Allicin Potential

The German Commission E recommends 4 g of fresh garlic, or equivalent, for therapeutic effects [65]. This is also the average size of a garlic clove [22,100] (p. 46). The average allicin potential of garlic cloves is 3.6 ± 0.9 mg/g fr wt or 10.8 mg/g dry wt [19,20,30,66]. The average alliin content of garlic cloves is 9.7 ± 2.0 mg/g fr wt or 27.7 ± 5.8 mg/g dry wt [49,50,51]. Almost all clinical trials with garlic powder supplements have used a daily dose standardized at 3.6–7.8 mg allicin potential from 7.8–17 mg alliin [1,2], which represents 1–2 g of typical raw garlic. It is here proposed that the minimum daily dose of a garlic powder supplement for possible health benefits should have high allicin bioavailability and represent the equivalent of two grams of typical raw garlic by having an allicin potential of about 7–8 mg or an alliin content of 18–21 mg (includes an additional 19% alliin for the other allyl thiosulfinates, Figure 1), and that the preferred dose for clinical trials should be two times this amount. A dose that is substantially higher than the equivalent of 4 g fresh garlic may be difficult for most people to tolerate. The amount of allicin potential or alliin in a supplement will depend upon their concentration in the garlic powder and the amount of garlic powder in the supplement. Section 3.5 describes the allicin potential and alliin content of the garlic powders in the supplements used in this study. Of these supplements, five of six enteric brands were standardized on allicin potential or alliin content, but only one brand of normal tablets and one brand of capsules were standardized as such (Table 1).

#### 4.6.2. Allicin Bioavailability and Dosage Form

It is recommended that the allicin bioavailability of garlic supplements should be at least 65%, based on in vivo tests. For normal (non-enteric) tablets, allicin bioavailability was consistently above 65% for all brands tested, and it was not significantly affected by meal type (Table 6). Allicin bioavailability among enteric tablets was above 65% for four out of five brands consumed with the low-protein meal, but it was strongly affected by meal type, with an average of only 37% for the three brands consumed with the high-protein meal. If an enteric tablet is to used, its allicin bioavailability should be validated and limitations on meal contents described. Although allicin bioavailability from capsules was unaffected by meal type, only one of three brands gave higher than 65% and this brand was uniquely made with a coarse garlic powder, which coarseness was probably responsible for its high value. Hence, capsules should not be used unless high allicin bioavailability has been demonstrated.

#### 4.6.3. Alliinase Activity

Although alliinase activity is essential to high allicin bioavailability, no standard for alliinase activity is being recommended. Supplements with activity over the broad range of 250–13,000 μg allicin min^−1^ g^−1^ garlic powder had high allicin bioavailability (Table 6), demonstrating that only minimal activity is required. In a prior study, the alliinase activity of 24 brands of enteric tablets was found to give a similar range, <100 to 14,000, with four brands giving less than 150 [30]. Because that study found low in vitro USP dissolution allicin release [29] to correlate with low alliinase activity, it was recommended that the minimum alliinase activity for supplements should be 4000 μg allicin min^−1^ g^−1^ garlic powder [30]; however, now that high allicin bioavailability has been found for supplements with activity as low as 250, the prior activity recommendation of 4000 is no longer valid.

## 5. Conclusions

The bioavailability of allicin from garlic powder supplements containing alliin and active alliinase can be as high as that from an equivalent amount of crushed raw garlic containing maximum allicin, when consumed with a meal. This was found to be true for all tested brands of normal (non-enteric) tablets, including the main garlic powder supplement, Kwai (N1), which has been used in a large number of clinical trials on the possible health benefits of garlic and validates the results of these trials as representing about 1 g of crushed raw garlic. Compared to non-enteric tablets, allicin bioavailability from enteric tablets was found to be much more variable (36–104%) and to be strongly reduced (22–57%) when consumed with a high-protein meal, due to delayed gastric emptying. The in vitro USP dissolution allicin release test was found to be unreliable for predicting allicin bioavailability from enteric tablets and to be only 20% accurate for predicting allicin bioavailability from non-enteric tablets and capsules, validating the importance of determining allicin bioavailability in vivo. Based on the allicin metabolite equivalence found for the 13 garlic powder supplements tested, a person would need to consume 0.7 to 27 tablets/capsules to obtain the same equivalence as consuming 2 g of crushed raw garlic; however, the recommended daily dose by the supplement manufacturers varied from 1–6 tablets/capsules per day, highlighting the need for more complete standardization. With a more correct understanding of allicin bioavailability, better standards are now being recommended for garlic powder supplements being used in future clinical trials and being consumed by the general public, which standards include a dosage form with sufficient allicin bioavailability and alliin content or allicin potential to represent a daily dose of 2–4 g of typical crushed raw garlic (Section 4.6).

Consumption of alliinase-inhibited cooked garlic was found to give higher than expected allicin bioequivalence, with AMS formation being about 30% (roasted garlic) or 16% (boiled garlic) that of crushed raw garlic. Hence, any health benefits found for raw garlic or garlic supplements representing raw garlic can probably be achieved by consuming higher amounts of cooked garlic, with the likely exception of antimicrobial effects in the gastrointestinal tract. The mechanism by which AMS can be formed when alliinase is inactive was explored. After selective removal of alliin and alliin-derived compounds from boiled garlic, allicin bioequivalence decreased by only about 50% (AFHG vs. PFHA), indicating that the body possesses the ability to partly metabolize G/SAC to AMS, an ability which is enhanced about 6-fold when alliinase is inhibited. The metabolism of alliin to AMS was found to be enhanced about 2-fold, in the absence of active alliinase, compared to consuming pure alliin. Using in-garlic response factors, it was found that alliin and G/SAC contribute about equally to the breath AMS found after consuming alliinase-inhibited cooked garlic, but when alliinase is active, such as for garlic supplements having high allicin bioavailability, G/SAC accounts for only about 5% of the AMS. Possible mechanisms for the metabolism of G/SAC and alliin to AMS in the absence of active alliinase have been proposed but not experimentally verified. Unanswered questions for future research on the metabolism of alliinase-inhibited garlic include (a) identity of the metabolic pathways from alliin and SAC to AMS when alliinase is inactive and (b) the mechanism by which the metabolism of alliin, and especially SAC, to AMS is enhanced when alliinase is inactive.

## Figures and Tables

**Table 1:** Garlic supplements: identification and claims for garlic powder content and standardization. ^1^

ID	Brand Name and Lot Number	Manufacturer	Garlic Powder Claim per Tablet or Capsule (g)	Weight of Tablet or Capsule ^2^ (g)	Standardization Claims (per Tablet or Capsule)	Daily Dose ^3^
Enteric coated tablets (all claim to be enteric coated)						
E1	Garlicin (584401)	Nature’s Way Products, Inc., Springville, UT, USA	0.35	0.66	3.2 mg allicin	1 × 2
E2	Garlique (09A301)	Chattem, Inc., Chattanooga, TN, USA	(0.40) ^4^	0.67	≥5.0 mg allicin	1 × 1
E3	Garlinase Fresh (95178189)	Enzymatic Therapy, Inc., Green Bay, WI, USA	0.32	0.48	11 mg alliin, 5.0 mg allicin	1 × 1
E4	Garlic-Gold (8097)	Olympian Labs, Inc., Scottsdale, AZ, USA	0.60	0.99	15.6 mg alliin, 7.2 mg allicin	1 × 1
E5	Sundown Naturals Odor-Free Garlic (239029-09)	Sundown, Inc., Boca Raton, FL, USA	0.40	0.74	none	1 × 2–3
E6	NOW Pure-Gar Garlic (104872)	NOW Foods, Glendale Heights, IL, USA (manufactured 1999)	0.60	0.90	5.0 mg allicin	1 × 1
Normal (non-enteric) tablets						
N1	Kwai forte 300 mg ^5^ (02051450)	Klosterfrau, Cologne, Germany	0.30	0.75	3.9 mg alliin, 1.8 mg allicin	1 × 3
N2	Odor Free Garlic (197348-04)	Nature’s Bounty, Inc., Bohemia, NY, USA	0.10	0.26	“contains allicin”	1 × 3–6
N3	Natural Brand Odorless Garlic 1000 (2522EJ1818)	General Nutrition Corp., Pittsburgh, PA, USA	1.00	1.80	none	1 × 1–2
N4	Kwai ^5^ (92021901)	Lichtwer Pharma, GmbH, Berlin, Germany (manufactured 1992)	0.10	0.27	1.3 mg alliin, 0.6 mg allicin	2 × 3
Capsules						
C1	Garlic (00839138)	Nature’s Sunshine Products, Inc., Spanish Fork, UT, USA	0.55 fine ^6^	0.67	none	1 × 2
C2	Deodorized Garlic 500 (2040348)	Vitamin Shoppe, Inc., North Bergen, NJ, USA (Pure-Gar garlic powder)	0.50 fine ^6^	0.69	none	1–3 ^3^
C3	GarliPure 500 mg (2037406)	Natrol, Inc., Chatsworth, CA, USA (Pure-Gar garlic powder)	0.50 coarse ^6^	0.68	5.0 mg alliin, 0.75 mg allicin ^7^	2 × 2

^1^ All supplements were manufactured in the U.S., except N1 and N4. ^2^ Average weight of at least 10 tablets or capsules (including shell). ^3^ Daily dose recommended on the product label. The first number is the number tablets or capsules recommended per meal; the second number is the number of times per day this amount is to be consumed. For example, 1 × 2–3 means to take 1 tablet two or three times per day. Product C2 simply stated to take 1–3 capsules per day. ^4^ For E2, no claim was made for the amount of garlic powder; it is estimated to be 0.40 g garlic powder per tablet, based on an average of 60% of tablet weight. ^5^ Kwai or Kwai Sapec or Kwai forte was originally made by Lichtwer Pharma, GmbH, Berlin, using 300 mg of LI 111 garlic powder that was standardized to contain 1.3% alliin and to yield 0.6% allicin. The tablets were used in numerous cardiovascular clinical trials from 1986 to 2005. In 2007, Lichtwer Pharma was sold to Klosterfrau Healthcare Group, Cologne, Germany, which has sold the same tablets under the names of Kwai forte 300 mg (apo-discounter.de) and Kwai Heartcare Garlic 300 mg (standardized allicin yield of 0.6% or 1.8 mg per tablet), with minor changes in the excipients. Kwai has also been sold in smaller tablets (100 mg garlic powder, product N4). ^6^ Sieving results. C1 contained fine, almost white particles: 91% smaller than 125 μm, 3% larger than 250 μm. C2 contained fine, almost white, and some darker particles: 85% smaller than 125 μm, 0.8% larger than 250 μm. C3 contained coarse, light brown and medium brown particles: 6% smaller than 125 μm, 79% larger than 250 μm. When C3 was purchased again (lot 2065372), seven years later (2016), the powder was much finer and appeared identical to product C2. ^7^ The claimed ratio of alliin to allicin is unusually high, 6.7. The normally claimed weight ratio of alliin to allicin is 2.2, representing a mole ratio of 2.0. However, the actual alliin to allicin weight ratio for garlic cloves and powders is about 2.5 because about 20% of the alliin forms other allyl thiosulfinates [19,20].

**Table 2:** Other ingredients in the garlic supplements, including possible enteric-coating agents: complete list, in the same order as on the label claims.

Supplement	Other Ingredients
Enteric coated	
E1 ^1^	cellulose, aqueous coating solution, modified cellulose gum, stearic acid ^2^, silica
E2	silicified microcrystalline cellulose, croscarmellose sodium, methacrylic acid, hypromellose, magnesium stearate, stearic acid ^2^, magnesium silicate, titanium dioxide, mineral oil, triacetin ^2^, polyethylene glycol ^2^, microcrystalline cellulose, glycerol monostearate, triethyl citrate ^2^, sodium lauryl sulfate
E3	cellulose, modified cellulose, modified cellulose gum, silica, titanium dioxide, calcium stearate, fractionated coconut oil, sodium alginate ^2^, vegetable glycerin, stearic acid ^2^
E4	enteric coating ^2^ (Eudagrit L30D-55, triethyl citrate, methylcellulose), microcrystalline cellulose, dicalcium phosphate, ascorbyl palmitate, modified cellulose, silica, stearic acid ^2^, corn starch
E5	calcium phosphate, cellulose, calcium carbonate, croscarmellose, cellulose coating ^2^, titanium dioxide, calcium silicate, food glaze, magnesium stearate, sodium alginate ^2^
E6 ^1^	calcium phosphate, cellulose, magnesium stearate, stearic acid ^2^, silica, enteric coating
Normal (non-enteric) tablets	
N1	lactose, cellulose, silica, magnesium stearate, castor oil, polyethylene glycol, hypromellose, sucrose, magnesium silicate, gelatin, povidone K25, carnauba wax, bleached wax, quinoline yellow E104, indigo carmine 132
N2	cellulose, dicalcium phosphate, titanium dioxide, silica, magnesium silicate, calcium carbonate, magnesium stearate, natural flavor
N3	calcium carbonate, cellulose, titanium dioxide, acetoglycerides
N4	lactose, cellulose, silica, magnesium stearate, sucrose, magnesium silicate, calcium carbonate, gum arabic, shellac, glucose, castor oil, bees wax
Capsules	
C1	gelatin, water
C2	gelatin, water, magnesium stearate, silicon dioxide
C3	gelatin, water, magnesium stearate

^1^ Products E1 and E6 do not list a main enteric coating agent. Later labels for product E1 (© 2013–© 2016) omitted the phrase “aqueous coating solution” and included sodium alginate, a common enteric coating agent; however, the newer tablets have a different appearance (less shine) than those used in this study, indicating a change in the coating formulation. ^2^ Known enteric coating agents. Stearic acid has additional purposes in tablet formulation. Only product E4 specifically stated which ingredients were used for the enteric coating.

**Table 3:** Summary of the experimental variation.

Source of Variation	Variation (RSD, %)	Measurement
Between triplicate injections of the same breath	3.4 (*n* = 12 sets)	Peak area
Between triplicate breaths taken 1 min apart	5.6 (*n* = 4 sets)	Peak area
Between 13 persons consuming the same product	42 (*n* = 21 sets) 32 (*n* = 21 sets)	AUC AUC_product_/AUC_control_
Between duplicate consumptions of a garlic product by the same person (within person)	17 (*n* = 39 sets)	AUC
Between duplicate sets of tests with 13 persons (between test sets)	4.7 (*n* = 3 sets)	Average AUC

RSD: relative standard deviation; AUC: area under the 32-h breath AMS concentration curve.

**Table 4:** Composition of the garlic products: *S*-allyl ^1^ compounds, as μmol of *S*-allyl per dose consumed.

Code or Name	Dose	Alliin ^2^	Allyl Thiosulfinates ^3^	Allyl Polysulfides ^4^	GSAC	SAC	AADD ^5^	TKSA ^6^
Control, raw homogenate	1.4 g ^7^	nd, <0.2	53.3 ^3^	1.4 ^4^	10.7	0.13	54.7	65.5
Garlic supplements								
Enteric tablets								
E1	2 tab	132 (33) ^2^	nd, <0.1	0.4	33.2	6.0	132	171
E2	2 tab	134 (30)	nd, <0.1	2.4	12.9	5.3	137	155
E3	3 tab	264 (49)	nd, <0.1	13.5 ^4^	29.3	16.4	278	323
E4	2 tab	174 (26)	nd, <0.1	5.8	63.6	10.6	180	254
E5	3 tab	44 (6.5)	nd, <0.1	7.3	62.7	6.8	52	121
E6	2 tab	106 (16)	nd, <0.1	3.9	41.0	5.8	110	157
Normal tablets								
N1	3 tab	72 (14)	nd, <0.1	3.8	48.5	4.1	75.8	128
N2	9 tab	40 (7.9) ^2^	nd, <0.1	5.6	52.9	4.3	45.2	102
N3	1 tab	155 (28)	nd, <0.1	0.5	44.7	7.7	155	207
N4	9 tab	57 (11)	nd, <0.1	2.4	47.4	6.4	58.8	113
Capsules								
C1	1 cap	73 (24)	nd, <0.1	0.2	28.4	5.1	73.0	106
C2	3 cap	167 (20)	nd, <0.1	2.5	97.7	14.1	169	281
C3	4 cap	98 (8.7)	nd, <0.1	1.9	77.5	26.1	99.5	203
Commercial garlic foods								
Pickled ^8^	12 g	388	nd, <0.2	9.0	32.6	25.7	397	455
Acid-minced	7 g	134	nd, <0.2	21 ^4^	81.9	14.5	155	251
Oil-chopped	2.5 g	36	nd, <0.2	36 ^4^	31.5	10.8	71.5	114
Black garlic	10.2 g	nd, <2	nd, <0.2	0.8	5.9	40.1	0.8	46.8
Kitchen-prepared garlic foods								
Roasted 160 °C	6.1 g	400	4.0	19.6 ^4^	92.7	5.4	423	521
Roasted 215 °C	5.5 g	400	0.4	7.9	82.0	8.6	409	499
Boiled 4 min	7.6 g	451	2.6	1.7	97.3	6.2	455	558
Boiled 45 min	5.5 g	450	0.2	2.2	86.4	0.9	452	540
Raw, diced	1.5 g	110	2.1	nd, <0.2	24.5	3.2	112	140
Special extracts ^9^								
PFHA	68 mL	399	nd, <0.1	0.05	68.7	2.7	399	470
AFHG	57 mL	nd, <0.4	nd, <0.1	nd, <0.03	63.6	7.0	nd, <0.5	70.6
AFHS	57 mL	nd, <0.4	nd, <0.1	nd, <0.03	3.4	67.2	nd, <0.5	70.6
AFHG + SAC	57 mL	nd, <0.4	nd, <0.1	nd, <0.03	63.6	148	nd, <0.5	212

^1^ Allicin and the diallyl polysulfides have two *S*-allyl groups per molecule; allyl methyl thiosulfinates, allyl methyl polysulfides, alliin, γ-glutamyl-*S*-allylcysteine (GSAC) and *S*-allylcysteine (SAC) have one *S*-allyl. ^2^ For alliin, a second set of values is included (in parentheses) for the supplements: mg alliin per gram garlic powder content claimed (Table 1); for product N2 the garlic powder content was not stated, but it was estimated (Table 1). ^3^ Allyl thiosulfinates = sum of allicin, allyl methyl thiosulfinates, and allyl-*trans*-1-propenyl thiosulfinates. Distribution for the control product, as mol % of *S*-allyl (allicin has two per molecule) and as mol % of compounds = allicin 80.3% and 67.1%, allyl methyl thiosulfinates 17.0% and 28.3%, and allyl-*trans*-1-propenyl thiosulfinates 2.7% and 4.6%. ^4^ Allyl polysulfides = sum of diallyl di-, tri- and tetrasulfides (DAS_2_, DAS_3_, DAS_4_) and allyl methyl di-, tri-, and tetrasulfides (AMS_2_, AMS_3_, AMS_4_). Diallyl sulfide (DAS) was typically <3% of the total allyl sulfides, and AMS was undetectable. Distribution for the control and the more abundant samples, as *S*-allyl mol %: control, DAS_2_ 72%, DAS_3_ 25%, AMS_3_ 3%; E3, DAS_2_ 22%, DAS_3_ 38%, DAS_4_ 22%, AMS_2_ 3%, AMS_3_ 10%, AMS_4_ 5%; Acid-minced, DAS_2_ 7%, DAS_3_ 69%, DAS_4_ 18%, AMS_2_ < 1%, AMS_3_ 4%, AMS_4_ 2%; Oil-chopped, DAS_2_ 28%, DAS_3_ 51%, DAS_4_ 8%, AMS_2_ 1%, AMS_3_ 10%, AMS_4_ 2%, ajoene 0.3%; Roasted 160 °C, DAS_2_ 16%, DAS_3_ 70%, DAS_4_ 8%, AMS_2_ < 1%, AMS_3_ 6%, AMS_4_ 1%. ^5^ AADD = alliin and alliin-derived dithioallyl compounds (alliin, allyl thiosulfinates, allyl polysulfides), expressed as μmol *S*-allyl per dose consumed. ^6^ TKSA = total known *S*-allyl compounds (AADD + GSAC + SAC), expressed as μmol *S*-allyl per dose consumed. ^7^ Control: 1.4 g of homogenate obtained from 0.88 g raw garlic (0.35 g dry wt). ^8^ The liquid in which the pickled garlic was immersed (35% of the container contents), although not normally consumed, contained substantial amounts of alliin (43% of the container contents), GSAC (43%), and SAC (41%), as the result of diffusion. ^9^ PFHA = protein-free, high alliin extract. AFHG = alliin-free, high GSAC extract. AFHS = alliin-free, high SAC extract.

**Table 5:** Breath AMS after consumption of the control (raw garlic homogenate) at the standard dose. ^1,2^

Test	Meal Protein	Mode	*n*	Tmax (h)	Cmax (ng/L)	% Cmax at 1 h ^3^	AUC ^4^ (ng-h/L)	RF (AUC/AADD)
1a	high	capsules	13	3.1 ± 1.0	54 ± 20	31 ± 28	351 ± 143	6.41 ± 2.62
1b	high	capsules	13	3.5 ± 1.1	47 ± 13	25 ± 16	332 ± 108	6.08 ± 1.97
1 av	high	capsules	13	3.3 ± 0.95 ^a^	51 ± 15 ^c^	28 ± 18 ^t^	342 ± 119 ^a^	6.25 ± 2.17 ^a^
2a	low	capsules	13	2.5 ± 0.6	69 ± 21	34 ± 21	399 ± 142	7.30 ± 2.60
2b	low	capsules	13	2.6 ± 0.6	69 ± 29	49 ± 26	406 ± 162	7.43 ± 2.97
2 av	low	capsules	13	2.6 ± 0.45 ^a^	68 ± 22 ^c^	41 ± 20 ^t^	403 ± 144 ^a^	7.37 ± 2.64 ^a^
2c ^2^	low	capsules	13	2.7 ± 0.8	51 ± 15	44 ± 24	342 ± 113	6.25 ± 2.07
2 final ^5^	low	capsules	13	2.6 ± 0.46 ^c^	63 ± 19 ^n^	42 ± 17 ^c^	383 ± 131 ^n^	6.99 ± 2.39 ^n,6^
3 ^7^	low	sandwich	13	1.4 ± 0.8 ^c^	60 ± 20 ^n^	88 ± 13 ^c^	400 ± 157 ^n^	7.31 ± 2.87 ^n^

^1^ Standard dose: 1.4 g homogenate from 0.88 g raw garlic (0.35 g dry wt); AADD = 54.7 μmol *S*-allyl. ^2^ Tests 1a to 2b (four tests) were conducted over a two month period. Tests 2a and 2b were conducted the same month. Test 2c was conducted nine months after tests 2a and 2b. Test 3 was conducted one month after test 2c. Values are mean ± SD (standard deviation). Statistical comparisons (share same letter) were made between (1) capsules consumed with high or low-protein meals (1 av vs. 2 av) and (2) consumption in capsules or sandwich for the low-protein meal (2 final vs. test 3). Designations for significance: n = not significant (*p* > 0.05); t = trend (*p* = 0.05–0.1), a = *p* < 0.05, c = *p* < 0.001. ^3^ The % Cmax at 1 h value provides an evaluation of Tmin; the true Tmin is often not known because the first breath sampled after consuming the product was usually taken at 1 h. ^4^ AUC = area under the 32 h concentration curve for breath AMS. ^5^ The standard control: average for all three tests conducted with the low-protein meal in capsules (2a, 2b, 2c). ^6^ The individual average response factor values (RF) for all 13 participants were, from low to high: 2.49, 4.37, 4.80, 4.93, 5.12, 7.31, 7.82, 7.97, 8.10, 8.72, 9.42, 9.90, 9.92 (av 6.99). ^7^ In test 3, the garlic homogenate was placed directly inside the bread and butter sandwich, immediately before consumption, rather than inside capsules.

**Table 6:** Allicin bioavailability for garlic supplements, based on breath AMS-AUC. ^1^

Product	Dose and AADD ^2^	Meal Protein	*n*	Alliinase Activity ^3^	Tmin ^4^ (h)	Tmax (h)	% Cmaxat 1 h	AUC (ng-h/L)	RF (AUC/AADD)	Allicin Bioavailability, RRF ^5^ (%)
Control ^2^	1.4; 54.7	low	13	-- ^3^	<1.1 ± 0.2	2.6 ± 0.5	42 ± 17	383 ± 131	7.0 ± 2.4 ^5^	100 ± 34
	1.4; 54.7	high	13	--	1.4 ± 0.3 ^b^	3.3 ± 0.95 ^a^	28 ± 18 ^a^	--	--	--
Enteric tablets										
E1	2; 132	low	12	12,650	2.6 ± 1.7 ^a^	4.7 ± 2.1 ^b^	20 ± 35 ^a^	802 ± 342	6.1 ± 2.6 ^n^	91 ± 28 ^n^
	2; 132	high	13		9.1 ± 6.5 ^bb^	10.3 ± 6.5 ^ba^	<1 ^ct^	496 ± 178	3.8 ± 1.4 ^cb^	57 ± 17 ^bb^
E2	2; 137	low	8	2360	2.2 ± 1.4 ^a^	4.1 ± 1.9 ^t^	27 ± 39 ^n^	782 ± 471	5.4 ± 3.1 ^a^	69 ± 29 ^a^
	2; 137	high	13		6.1 ± 2.4 ^cc^	7.1 ± 3.1 ^ca^	<1 ^ct^	324 ± 205	2.7 ± 1.6 ^ct^	33 ± 16 ^ca^
E3	3; 278	low	4	1950	<1.0 ± 0.1 ^n^	2.6 ± 1.7 ^n^	50 ± 34 ^n^	720 ± 235	2.7 ± 0.7 ^a^	36 ± 12 ^a^
	3; 278	high	7		2.6 ± 0.8 ^ab^	4.9 ± 2.0 ^tn^	11 ± 29 ^nn^	432 ± 192	1.6 ± 0.7 ^ca^	22 ± 7 ^ba^
E4	2; 180	low	7	13,500	<1.0 ± 0.1 ^n^	3.3 ± 1.2 ^n^	38 ± 26 ^n^	826 ± 473	4.6 ± 2.6 ^n^	71 ± 33 ^n^
E5	3; 51.7	low	7	520	<1.4 ± 0.5 ^n^	3.6 ± 1.4 ^n^	35 ± 30 ^n^	407 ± 156	7.9 ± 3.0 ^n^	104 ± 31 ^n^
E6 ^6^	2; 106	low	3	550	4.6 ± 0.5 ^a^	6.3 ± 0.6 ^a^	0.8 ± 1.9 ^c^	496 ± 160	4.7 ± 1.5 ^a^	52 ± 12 ^b^
Normal tablets										
N1	3; 75.8	low	13	6520	<1.3 ± 0.3 ^t^	3.0 ± 1.0 ^n^	47 ± 29 ^n^	400 ± 137	5.3 ± 1.8 ^b^	80 ± 26 ^n^
	3; 75.8	high	13		1.6 ± 0.5 ^nn^	3.9 ± 1.1 ^aa^	12 ± 13 ^ab^	385 ± 169	5.1 ± 2.2 ^cn^	73 ± 17 ^an^
	3; 75.8	high-w ^7^	13		1.5 ± 0.4 ^nn^	3.9 ± 1.4 ^nn^	27 ± 32 ^nt^	457 ± 230	6.0 ± 3.0 ^nn^	85 ± 33 ^nn^
N2	9; 41.5	low	13	360	<1.2 ± 0.3 ^n^	2.1 ± 0.6 ^b^	66 ± 27 ^a^	294 ± 135	6.5 ± 3.0 ^n^	93 ± 32 ^n^
	9; 41.5	high	13		<1.3 ± 0.5 ^nn^	3.5 ± 0.9 ^nc^	18 ± 14 ^tc^	263 ± 128	5.8 ± 2.8 ^an^	82 ± 30 ^nn^
N3	1; 155	low	4	10,700	<0.9 ± 0.1 ^n^	2.2 ± 0.5 ^n^	47 ± 19 ^n^	1247 ± 357	8.0 ± 2.3 ^n^	111 ± 38 ^n^
N4 ^6^	9; 58.8	low	4	1160	1.6 ± 0.6 ^t^	3.9 ± 0.6 ^a^	8 ± 12 ^a^	345 ± 168	5.9 ± 2.9 ^n^	76 ± 31 ^n^
Capsules										
C1	1; 73	low	13	10,800	<1.0 ± 0.3 ^n^	2.3 ± 0.7 ^t^	51 ± 24 ^n^	267 ± 96	3.7 ± 1.3 ^c^	54 ± 14 ^b^
	1; 73	high	12		<1.2 ± 0.4 ^nn^	3.6 ± 0.6 ^ac^	22 ± 14 ^nb^	281 ± 132	3.9 ± 1.8 ^cn^	53 ± 13 ^cn^
C2	3; 169	low	13	210	1.5 ± 0.5 ^a^	3.6 ± 1.2 ^a^	19 ± 24 ^b^	290 ± 119	1.7 ± 0.7 ^c^	26 ± 11 ^c^
	3; 169	high	13		2.2 ± 0.7 ^ba^	4.8 ± 2.0 ^aa^	2 ± 6 ^ca^	314 ± 123	1.9 ± 0.7 ^cn^	27 ± 8 ^cn^
C3	4; 99.5	high	13	250	<1.3 ± 0.8 ^n^	3.8 ± 1.0 ^t^	6 ± 4 ^b^	733 ± 245	7.4 ± 2.5 ^n^	109 ± 24 ^n^

^1^ Values are mean ± SD for *n* persons. For Tmin, Tmax and % Cmax at 1 h, statistical comparisons were made between (1) products and the control of the same meal type (first set of designators) and (2) the high-protein and low-protein meal tests for the same product, when both were conducted (second set). For RF and allicin bioavailability, the first set of comparisons was made between the products and the standard, low-protein, control; the second set was made between meal types of the same product. Unpaired *t*-Test was used in three cases, where *n* was 3–4. Designations for significance: n = not significant (*p* > 0.05); t = trend (*p* = 0.05–0.1) a = *p* < 0.05, b = *p* < 0.01, c = *p* < 0.001. ^2^ The dose consumed is expressed as: (left) the number of tablets or capsules or the weight of the control and (right) the AADD value (see Table 4). Control: 1.4 g of homogenate, made from 0.88 g raw garlic. ^3^ Alliinase activity is expressed as μg allicin min^−1^ g^−1^ garlic powder content. When the control was prepared, the alliinase activity was sufficient to rapidly convert all of the alliin to allyl thiosulfinates. ^4^ Tmin = the time (h) after consumption until AMS was first detected in the breath, with the limitation that the first breath was taken at about 1 h after consumption. ^5^ Allicin bioavailability = RRF = 100% × (RF_product_/RF_control_). The RF_control_, determined with the low-protein meal (standard control, see Table 5), was used for all determinations, regardless of product meal type. ^6^ Products E6 and N4 were consumed 12 and 19 years, respectively, after their manufacture; they were stored at ambient temperature. Nine tablets of N4 contained the same amount of garlic powder as three tablets of N1. ^7^ Extra water (225 mL) was consumed with the high-protein meal (high-w).

**Table 7:** Gender comparisons for Tmax upon consumption of enteric-coated tablets with high-protein (HP) and low-protein (LP) meals.

Product	Meal	Female			Male			Female:Male	
		*n*	Tmax (h)	Range (h)	*n*	Tmax (h)	Range (h)	*p*-Value	Ratio
E1	HP	6	14.5 ± 7.3	6.1–24	7	6.6 ± 2.6	3.0–11	0.04	2.2
E1	LP	6	7.2 ± 5.5	4.9–18	7	4.2 ± 2.5	1.2–8.4	>0.2	1.7
E2	HP	6	9.5 ± 2.8	5.3–16	7	5.1 ± 1.5	3.1–6.7	0.004	1.9
E2	LP	4	4.5 ± 2.0	2.1–7.0	4	3.7 ± 1.8	1.1–5.3	>0.2	1.2

Values are mean ± SD. *p*-Values are based on the *t*-test with unequal variances.

**Table 8:** Comparison between USP dissolution allicin release (in vitro) and allicin bioavailability (in vivo) for garlic supplements.

Product	Alliinase Activity ^2^	Potential Allicin ^3^ (mg; % of Claim)	Dissolution Results (In Vitro)				In Vivo Results (LP meal) ^1^		
			Disinteg Ration Time ^4^ (h)	Allicin Release ^5^ (mg)	Allicin Release ^6^ (%)	Allicin Release ^7^ (% of Claim)	Allicin Bioavailability, RRF (%)	Ratio: ^8^ In Vivo/In Vitro	Tmax (h)
Enteric tablets (rapid in vitro disintegration)									
E1	12,650	3.92 (123)	2 + 1	3.37	86	105	91	1.1	4.7
E2	2360	4.20 (84)	2 + 0.75	3.90	93	78	69	0.7	4.1
E5	520	0.85 (nc)	2 + 1	0.71	84	nc	104	1.2	3.6
Enteric tablets (slow in vitro disintegration)									
E3	1950	5.17 (103)	2 + 2.5	0.13	3 (12) ^9^	3	36	12	2.6
E4	13,500	5.45 (76)	2 + 5.7	0.33	6 (13) ^9^	5	71	12	3.3
E6	550	3.31 (66)	2 + 3.5	0.21	6 (28) ^9^	4	52	9	6.3
Normal tablets									
N1	6520	1.65 (92)	1.5 + 0	0.20	12	13	80	7	3.0
N2	360	0.27 (nc)	1.75 + 0	0.015	6	nc	93	16	2.1
N3	10,700	11.1 (nc)	1.75 + 0	1.84	17	nc	111	7	2.2
Capsules ^1^									
C1	10,840	5.0 (nc)	2 + 1	2.1	42	nc	54	1.3	2.3
C2	210	3.08 (nc)	2 + 0	<0.03	<1	nc	26	>27	3.6
C3	250	1.27 (170)	0.25 + 0	<0.03	<1	<4	109	>109	3.8

^1^ The in vivo results for allicin bioavailability (RRF) and Tmax are from Table 6. All products were consumed with the low-protein (LP) meal, except C3 was consumed with the high-protein (HP) meal. ^2^ Alliinase activity is expressed as μg allicin min^−1^ g^−1^ garlic powder content. ^3^ Potential allicin (allicin potential): allicin found (mg/tablet or capsule) after pulverized tablets or capsule contents were stirred in water for 30 min; it is also given as % of label claim reported in Table 1, except when there was no claim (nc) for allicin. ^4^ Time to achieve complete disintegration in the dissolution test. The first 2 h was in acid; the remaining time was in buffer. Values are the average of two tests. ^5^ Dissolution allicin release (mg): allicin found (mg/tablet or capsule) at the United States Pharmacopeia (USP) standard time of 1 h in buffer, after 2 h in acid. Values are the average of two tests. ^6^ Dissolution allicin release (%): allicin found, as percent of potential allicin, at the USP standard time of 1 h in buffer, after 2 h in acid. ^7^ Dissolution allicin release (% of claim): dissolution allicin release (mg) compared to label claim (Table 1), except when there was no claim (nc). ^8^ Ratio (in vivo/in vitro): allicin bioavailability (%)/dissolution allicin release (%). ^9^ Dissolution allicin release (%), at the time of complete disintegration, when greater than 1 h in buffer.

**Table 9:** Kitchen-prepared and commercial garlic foods: allicin bioequivalence. ^1,2^

Garlic Food	Dose and AADD ^3^	*n*	Alliinase Activity ^4^	Tmax (h)	% Cmax at 1 h	AUC (ng-h/L)	Predicted AUC ^5^ and Ratio: Actual/Predicted	RF (AUC/AADD)	Allicin Bioequivalence (AADD, RRF) ^6^ (%)	Allicin Bioequivalence (TKSA, RRF2) ^7^ (%)
Control ^8^	1.4; 54.7	7a ^8^	--	1.8 ± 1.4 ^8^	87 ± 14 ^8^	396 ± 147	--	7.23 ± 2.68	100 ± 37	100±37
Control	1.4; 54.7	7b	--	1.3 ± 0.8	91 ± 12	392 ± 129	--	7.17 ± 2.35	100 ± 33	100±33
Kitchen-prepared garlic foods (alliinase-inhibited, except raw, diced)										
Roasted 160 °C	6.1; 423	7b	nd	4.2 ± 1.0 ^b^	30 ± 15 ^c^	955 ± 214	169 (6)	2.3 ± 0.5 ^b^	34 ± 11 ^b^	33 ± 11 ^b^
Roasted 215 °C	5.5; 409	7b	nd	3.6 ± 1.1 ^bn^	27 ± 8 ^cn^	773 ± 191	64 (12)	1.9 ± 0.5 ^bn^	27 ± 5 ^bn^	27 ± 5 ^bn^
Boiled 4 min	7.6; 455	7a	nd	4.2 ± 1.0 ^b^	19 ± 6 ^c^	580 ± 209	31 (19)	1.3 ± 0.5 ^c^	18 ± 2 ^b^	17 ± 2 ^b^
Boiled 45 min	5.5; 452	7a	nd	3.8 ± 1.2 ^an^	26 ± 6 ^cn^	428 ± 116	17 (25)	1.0 ± 0.3 ^ca^	14 ± 4 ^ba^	14 ± 4 ^ba^
Raw, diced ^1^	1.5; 112	7b	19,000	3.1 ± 0.9 ^b^	53 ± 20 ^a^	618 ± 207	803 (0.8)	5.5 ± 1.8 ^t^	80 ± 28 ^n^	77 ± 27 ^n^
Commercial garlic foods (alliinase-inhibited)										
Pickled	12; 397	7a	nd	3.1 ± 0.8 ^n^	20 ± 8 ^c^	560 ± 211	65 (9)	1.4 ± 0.5 ^c^	19 ± 2 ^b^	21 ± 3 ^b^
Acid-minced	7; 155	7b	nd	3.7 ± 0.5 ^c^	31 ± 14 ^c^	717 ± 227	153 (5)	4.6 ± 1.5 ^b^	66 ± 15 ^b^	49 ± 11 ^a^
Oil-chopped	2.5; 71.5	7a	nd	4.4 ± 1.3 ^a^	21 ± 9 ^c^	410 ± 169	260 (1.5)	5.7 ± 2.4 ^a^	79 ± 14 ^t^	59 ± 10 ^a^
Black garlic	10.2; 0.8	4	nd	3.2 ± 0.6 ^t^	34 ± 8 ^b^	228 ± 72	6 (39)	-- ^9^	-- ^9^	5.0 ± 0.4 ^c,9^

^1^ All garlic foods were consumed with the low-protein meal, inside the sandwich. For raw, diced garlic, the allicin bioequivalence values represent allicin bioavailability because it has active alliinase. ^2^ Values are mean ± SD for *n* persons. Statistical comparisons were made between the foods and the controls (first set of designators) and between the intensity of cooking for the same kitchen food (boiled, roasted; second set). Designations for significance: n = not significant (*p* > 0.05); t = trend (*p* = 0.05–0.1), a = *p* < 0.05, b = *p* < 0.01, c = *p* < 0.001. ^3^ The dose consumed is expressed as: (left) the weight, in g, of the product and (right) the AADD value. ^4^ Alliinase activity: when none was detected (nd), the detection limit was <1. ^5^ The predicted AMS-AUC is based only on the content of allyl thiosulfinates and allyl polysulfides (Table 4) times the average RF for the raw homogenate control (7.23 or 7.17); for diced raw garlic, it also includes alliin because active alliinase is present. ^6^ Allicin bioequivalence (AADD) = RRF = 100% × (RF_product_/RF_control_). ^7^ Allicin bioequivalence (TKSA) = RRF2 = 100% × (RF2_product_/RF2_control_). RF2 = AUC/TKSA; RF2_control_ = 394/65.5 = 6.02. ^8^ Control: 1.4 g of homogenate from 0.88 g raw garlic. The control values for two groups of seven persons (7a, 7b, with one person common to both groups) were matched to the individuals who consumed the garlic foods. The values for Tmax and % Cmax at 1 h are for the “in sandwich” control (Table 5, test 3). ^9^ Black garlic: allicin bioequivalence was determined on product weight basis, RRF3. RRF3 = 100% × (RF3_product_/RF3_control_). RF3 = AUC/wt consumed. RF3_product_ = 228/10.2. RF3_control_ = 394/0.88. RRF3 = 100% × 22.4/448 = 5.0%.

**Table 10:** Special garlic extracts and pure compounds: allicin bioequivalence. ^1,2^

Name	Dose	*n*	Alliin (μmol)	GSAC (μmol)	SAC (μmol)	TKSA (μmol)	Tmax (h)	AUC (ng-h/L)	RF2 (AUC/TKSA)	Allicin Bioequivalence (TKSA, RRF2) (%)
Control	1.4 g ^3^	4	nd, <0.2 ^3^	10.7	0.13	65.5	1.6 ± 0.6 ^4^	446 ± 116	6.8 ± 1.8	100 ± 26
Control	1.4 g	1 (3)	nd, <0.2	10.7	0.13	65.5	1.6 ± 0.9	543 ± 44	8.3 ± 0.7	100 ± 8
Special extracts ^5^										
PFHA	68 mL	4	399	68.7	2.7	470	3.6 ± 1.3 ^t^(vs. AFHG) ^n^	472 ± 78	1.01 ± 0.18 ^b^(vs. AFHG) ^b^	14.7 ± 2.6 ^b^(vs. AFHG) ^b^
AFHG	57 mL	4	nd, <0.5	63.6	7.0	70.6	2.9 ± 1.5 ^t^	203 ± 15	2.85 ± 0.17 ^a^	42 ± 3 ^a^
AFHG	57 mL	1 (2)	nd, <0.5	63.6	7.0	70.6	2.3 ± 0.4 ^n^	212 ± 4	3.01 ± 0.06 ^b^	36.4 ± 0.7 ^b^
AFHS	57 mL	1 (2)	nd, <0.5	3.4	67.2	70.6	2.5 ± 0.7 ^n^(vs. AFHG) ^n^	216 ± 32	3.05 ± 0.45 ^b^(vs. AFHG) ^n^	36.8 ± 5.5 ^b^(vs. AFHG) ^n^
AFHG + SAC	57 mL + 141 μmol	1 (2)	nd, <0.5	63.6	148	212	3.5 ± 0.7 ^n^(vs. AFHG) ^n^	277 ± 1.4	1.31 ± 0.01 ^b^(vs. AFHG) ^c^	15.8 ± 0.1 ^b^(vs. AFHG) ^c^
Pure compounds ^6^										
Alliin, l(+)	373 μmol	1 (2)	373	--	--	373	3.2 ± 0.3 ^n^	143 ± 21	0.38 ± 0.06 ^c^	4.6 ± 0.7 ^c^
*S*-allyl-cysteine	676 μmol	1 (3)	--	--	676	676	8.4 ± 1.3 ^b,4^(vs. alliin) ^a^	333 ± 34	0.49 ± 0.05 ^b^(vs. alliin) ^n^	6.0 ± 0.6 ^b^(vs. alliin) ^n^
Diallyl sulfide	696 μmol	1 (3)	--	--	--	696^4^	5.8 ± 1.1 ^a,4^(vs. alliin) ^t^(vs. SAC) ^t^	188 ± 84	0.27 ± 0.12 ^b^(vs. alliin) ^n^(vs. SAC) ^a^	3.3 ± 1.5 ^b^(vs. alliin) ^n^(vs. SAC) ^a^

^1^ All products were consumed with the low-protein meal, as liquids (PFHA, AFHG, AFHG + SAC, AFHS) or inside the sandwich (alliin) or in capsules (control, *S*-allylcysteine (SAC), diallyl sulfide). ^2^ Values are mean ± SD for *n* persons. The control, PFHA and AFHG were consumed by the same four persons. When *n* is 1, it was the same person; the number in parentheses is the number of tests for that person. Statistical comparisons were made between the products and the controls and (in parentheses) between designated products. Unpaired *t*-tests were used for products consumed by only one person. Designations for significance: n = not significant (*p* > 0.05), t = trend (*p* = 0.05–0.1), a = *p* < 0.05, b = *p* < 0.01, c = *p* < 0.001. ^3^ Control: 1.4 g of homogenate from 0.88 g raw garlic. It contained no alliin, but it contained 54.7 AADD (Table 4). ^4^ The control value for Tmax is the “in sandwich” control (Table 5, Test 3), closely matching how the extracts were consumed. For *S*-allylcysteine and diallyl sulfide (consumed in capsules), the matched control Tmax value is 2.8 ± 0.3 h. ^5^ Special extracts. PFHA: protein-free, high alliin (high GSAC) extract. AFHG: alliin free, high GSAC extract. AFHS: alliin-free, high SAC (low GSAC) extract. AFHG + SAC: AFHG plus added SAC. For composition, see Table 4. None of these extracts contain active alliinase. ^6^ Purity: 99%, Section 2.1.

**Table 11:** Accounting for the AMS-AUC values for alliinase-inhibited garlic foods.

Garlic Food	Predicted Contribution to the AUC ^1^ (and % of Actual AUC)				AUC ^1^ (ng-h/L)
	Alliin ^2^	AllylTS/Sx ^3^	G/SAC ^4^	Total	
Response factor	0.67 ^2^	7.2 ^3^	2.85 ^4^		
Roasted 160 °C	268 (28)	168 (18)	280 (29)	716 (75)	955
Roasted 215 °C	268 (35)	60 (8)	258 (33)	586 (76)	773
Boiled 4 min	302 (52)	31 (5)	295 (51)	628 (108)	580
Boiled 45 min	302 (71)	17 (4)	249 (58)	569 (133)	428
Pickled garlic	260 (46)	65 (12)	166 (30)	491 (88)	560
Acid-minced	90 (13)	153 (21)	275 (38)	518 (72)	717
Oil-chopped	24 (6)	259 (63)	121 (30)	404 (99)	410
Average (%)	(36)	(19)	(39)	(93)	

^1^ Predicted AMS-AUC (ng-h/L) for each compound, calculated as the in-garlic response factor times μmol consumed. For μmol consumed, see Table 4. The actual AUC values for seven persons each is given in Table 9. ^2^ Alliin. The in-garlic RF_alliin_ has been calculated based on the AUC for PFHA, where 85% of the TKSA is alliin, minus the AUC contribution from G/SAC. Calculation: RF_alliin_ = (AUC_PFHA_ − AUC_G/SAC_)/μmol alliin; AUC_PFHA_ = 472 (Table 10); AUC_G/SAC_ = RF_G/SAC_ (2.85) × 71.4 μmol = 203; RF_alliin_ = (472 − 203)/μmol alliin (399) = 0.67. ^3^ AllylTS/Sx (allyl thiosulfinates and allyl polysulfides). This is the same RF as for the raw homogenate control (7.2, Table 9), where all of the alliin had been converted to AllylTS/Sx and where G/SAC account for only 1–8% of the AUC. ^4^ G/SAC (GSAC + SAC). The in-garlic RF_G/SAC_ (2.85) is the same as the RF for AFHG (Table 10, *n* = 4), where >99% of the TKSA is due to G/SAC.

**Table 12:** Accounting for the AMS-AUC values for garlic supplements having active alliinase.

Product and Meal	RRF (%)	AUC (ng-h/L)	Rf_alliin_^1^		% of Alliin to AMS ^2^		% Contribution to AMS-AUC					
							Alliin ^3^	Allyl Polysulfides ^4^	Alliin + Allyl Polysulfides		G/SAC ^5^	
RF_G/SAC_^6^			0.49	2.0	0.49	2.0	0.49	0.49	0.49	2.0	0.49	2.0
N3 (LP)	111	1247	7.9		109		98	<2	98		2	
C3 (HP)	109	733	6.8		95		93	<2	93		7	
E5 (LP)	104	407	7.2		100		79	13	92		8	
N2 (LP)	93	294	5.7		79		76	14	90		10	
E1 (LP)	91	802	5.9		82		98	<2	98		2	
N1 (LP)	80	400	4.8		67		86	7	93		7	
E4 (LP)	71	826	4.3		60		91	5	96		4	
E2 (LP)	69	782	5.6		78		97	2	99		1	
E1 (HP)	57	496	3.6		50		96	<2	96		4	
C1 (LP)	54	267	3.4		48		94	<2	94		6	
E3 (LP)	36	720	2.3	2.0	32	28	84	13	97	87	3	13
E2 (HP)	33	324	2.4	2.0	31	28	92	5	97	89	3	11
C2 (LP)	26	290	1.3	0.3	18	4	75	6	81	23	19	77
E3 (HP)	22	432	1.2	0.9	16	13	73	22	95	79	5	21

^1^ The RF_alliin_ value needed to obtain the observed AUC = (AUC − AUC_allyl polysulfides_ − AUC_G/SAC_)/μmol alliin. AUC_allyl polysulfides_ = RF_allyl polysulfides_ (7.2) × μmol allyl polysulfides; AUC_G/SAC_ = RF_G/SAC_ (0.49 or 2.0) × μmol G/SAC; amounts of alliin, allyl polysulfides, and G/SAC are in Table 4. ^2^ Percent of the alliin in the product that has been metabolized to AMS through garlic’s alliinase activity = 100 × RF_alliin_ divided by the RF value for alliin (7.2) when alliinase is not inhibited. The small amount of alliin metabolized to AMS independent of alliinase (RF_alliin_ < 0.67) has not been included. ^3^ Percent of breath AMS-AUC that comes from alliin = RF_alliin_ × μmol_alliin_ consumed × 100/AUC. ^4^ Percent of breath AMS-AUC that comes from allyl polysulfides = RF_allyl polysulfides_ × μmol_allyl polysulfides_ consumed × 100/AUC. ^5^ Percent of breath AMS-AUC that comes from G/SAC = RF_G/SAC_ (0.49 or 2.0) × μmol_G/SAC_ consumed × 100/AUC. ^6^ Response factors for G/SAC. The value of 0.49 is based on consumption of pure SAC. The value of 2.0 represents the estimated enhanced in-garlic response factor when alliinase is mostly, but probably not completely, inhibited in vivo; it is applied here only when the allicin bioavailability is less than 40%.

**Table 13:** Equivalence among garlic products, based on the allicin metabolite, AMS.

Product and Meal	Dose ^1^	Garlic Powder ^2^ (g)	Alliin ^3^ (μmol)	AUC (ng-h/L)	RRF (%)	Dose Equivalent to 2 g Raw Garlic ^4^	Dose Equivalent to 3 N1 Tabs ^5^
Raw, control	1.0 g ^1^	(0.40) ^2^	(61) ^3^	435	100	2.0	0.92
Garlic supplements—Enteric tablets							
E1 (LP)	2 tab	0.70	132	802	91	2.2	1.0
E1 (HP)	2 tab	0.70	132	496	57	3.5	1.6
E2 (LP)	2 tab	0.80	134	782	69	2.2	1.0
E2 (HP)	2 tab	0.80	134	324	33	5.4	2.5
E3 (LP)	3 tab	0.96	264	720	36	3.6	1.7
E3 (LP)	3 tab	0.96	264	432	22	6.0	2.8
E4 (LP)	2 tab	1.2	174	826	71	2.1	1.0
E5 (LP)	3 tab	1.2	44	407	104	6.4	2.9
E6 (LP)	2 tab	1.2	106	496	52	3.5	1.6
Garlic supplements—Normal tablets							
N1 (LP)	3 tab	0.90	72	400	80	6.5	3.0
N2 (LP)	9 tab	0.90	40	294	93	27	12
N3 (LP)	1 tab	1.0	155	1247	111	0.7	0.3
N4 (LP)	9 tab	0.9	57	345	76	23	10
Garlic supplements—Capsules							
C1 (LP)	1 cap	0.55	73	267	54	3.3	1.5
C2 (LP)	3 cap	1.5	167	290	26	9.0	4.1
C3 (HP)	4 cap	2.0	98	733	109	4.7	2.2
Garlic foods ^6^							
Roasted 160 °C	6.1 g	--	400	955	34	5.6	2.6
Roasted 215 °C	5.5 g	--	400	773	27	6.2	2.8
Boiled 4 min	7.6 g	--	451	580	18	11	5.2
Boiled 45 min	5.5 g	--	450	428	14	11	5.1
Raw, diced	1.5 g	--	110	618	80	2.1	1.0
Pickled	12 g	--	388	560	19	19	8.6
Acid-minced	7 g	--	134	717	66	8.5	3.9
Oil-chopped	2.5 g	--	36	410	79	5.3	2.4

^1^ Dose consumed, expressed as number of tablets or capsules or weight of garlic foods. For the raw garlic control, the dose is 1.0 g of clove, from which the homogenate was made, not 1.0 g of homogenate. ^2^ Amount of garlic power claimed in the supplement dose consumed (see Table 1). For the raw garlic control, it represents the dry weight of the cloves (0.395 g dry wt/g fr wt). ^3^ Amount of alliin found in the consumed dose (Table 4). For the raw garlic control, all the alliin was transformed to allyl thiosulfinates upon homogenization; the value shown (61 μmol or 10.8 mg/g fr wt) is the alliin that was present before homogenization, calculated from the allyl thiosulfinates (Section 3.5). ^4^ The dose of garlic product (number of tablets/capsules or grams of garlic foods) needed to obtain the same AMS-AUC that is found after consuming 2 g of the raw garlic used as the control = (dose of product consumed) × (AUC for 2 g of raw garlic, 870 ng-h/L)/(AUC of the product). ^5^ The dose of garlic product needed to obtain the same AMS-AUC that is found after consuming three N1 tablets = (dose of product consumed) × (AUC for three N1 tablets, 400 ng-h/L)/(AUC of the product). ^6^ Garlic foods were all consumed with the LP meal.
